# Stimulus specificity in combined action observation and motor imagery of typing

**DOI:** 10.1177/17470218241241502

**Published:** 2024-04-10

**Authors:** Camilla Woodrow-Hill, Emma Gowen, Stefan Vogt, Eve Edmonds, Ellen Poliakoff

**Affiliations:** 1 Division of Psychology, Communication and Human Neuroscience, The University of Manchester, Manchester, UK; 2 Psychology Department, Lancaster University, Lancaster, UK

**Keywords:** Action observation, motor imagery, keyboard typing, AO + MI, movement performance, motor simulation

## Abstract

Combined action observation and motor imagery (AO + MI) can improve movement execution (ME) in healthy adults and certain patient populations. However, it is unclear how the specificity of the observation component during AO + MI influences ME. As generalised observation could result in more flexible AO + MI rehabilitation programmes, this study investigated whether observing typing of target words (specific condition) or non-matching words (general condition) during AO + MI would have different effects on keyboard typing in healthy young adults. In Experiment 1, 51 students imagined typing a target word while watching typing videos that were either specific to the target word or general. There were no differences in typing execution between AO + MI conditions, though participants typed more slowly after both AO + MI conditions compared with no observation or imagery. Experiment 2 repeated Experiment 1 in 20 students, but with a faster stimulus speed in the AO + MI conditions and increased cognitive difficulty in the control condition. The results showed that the slowed typing after AO + MI was likely due to a strong influence of task-switching between imagery and execution, as well as an automatic imitation effect. Both experiments demonstrate that general and specific AO + MI comparably affect ME. In addition, slower ME following both AO + MI and a challenging cognitive task provides support for the motor-cognitive model of MI.

## Public significance statement

Across two experiments, we show that when simultaneously watching and imagining movement to influence physical movement, the specificity of the action observed does not differently affect physical movement in the context of keyboard typing. In addition, these results show that increased cognitive effort can decrease movement speed, and that people may automatically imitate the typing speed of an observed actor regardless of their intention.

## Introduction

Action observation (AO) is the act of watching movements performed by others (Hardwick et al., 2018), while motor imagery (MI) is the effortful process of mentally rehearsing oneself moving without physical movement (Jeannerod, 1994). Both AO and MI activate the premotor cortex and supplementary motor area (SMA), which are also involved in the execution of physical movement (Hardwick et al., 2018). Importantly, both AO and MI have been shown to influence physical movement execution (ME). When the observed or imagined action is the same or congruent with the action to be executed, performance elements can be improved, such as speed (Brass et al., 2000; Debarnot et al., 2011), accuracy (Guillot et al., 2015), and strength (Fontani et al., 2007), but when the observed/imagined actions are incongruent with the executed action, such as imagining and executing grasping of non-matching objects, ME can be hindered (Ramsey et al., 2010).

Congruent AO and MI have the potential to improve ME in people recovering from stroke (Kuk et al., 2016; B. Zhang et al., 2019) or in people with Parkinson’s disease (Agosta et al., 2017; Tamir et al., 2007). However, combining AO and MI (i.e., watching a movement while imagining oneself perform it; AO + MI) may have more influence on ME than either AO or MI alone. Greater activation of the motor areas of the brain has been evidenced during AO + MI compared with either AO or MI alone (Berends et al., 2013; Eaves, Behmer, & Vogt, 2016; Eaves, Riach, et al., 2016), and there is evidence for greater influence on ME resulting from extended AO + MI training programmes, such as improved dart-throwing ability (Romano-Smith et al., 2019). Although ME benefits are seen with extended AO + MI-based practise, short-term priming effects are also evidenced in the form of greater imitation when the AO and MI components are compatible compared with incompatible components (Eaves et al., 2014). While much of the existing AO + MI literature has focused on larger, gross movements (e.g., Marusic et al., 2018; Scott et al., 2018), there is preliminary evidence for AO + MI also improving dexterous actions (e.g., Bek et al., 2021; Sun et al., 2016). However, owing to the small samples used in some of these studies (e.g., Sun et al., 2016) and their focus on a specific patient population, more research is required.

Whether the AO and MI components in AO + MI are congruent differentially influences ME (Eaves et al., 2014). When the observed and imagined actions are the same (congruent AO + MI), there is greater corticospinal excitability, but when they conflict (incongruent AO + MI), movement-evoked potentials are reduced (Bruton et al., 2020). However, it is worth noting that in Bruton and colleagues (2020), the incongruent condition involved imagining a static hand, which would require no movement, and so may automatically reduce corticospinal excitability regardless of congruency. In addition, a third type, *coordinative* AO + MI, appears to influence ME somewhere between congruent and incongruent AO + MI (Bruton et al., 2020). Coordinative AO + MI occurs when the actions observed and imagined are not the same but share a similarity, such as the plane of motion, direction, or temporal pace (Bruton et al., 2020; Eaves et al., 2022; Vogt et al., 2013). In certain contexts, coordinative AO + MI may even be more beneficial than congruent AO + MI, such as watching an opponent’s fencing attacks while imagining oneself defending (see Eaves et al., 2022 for review). Dual-action simulation theory posits that AO and MI form two parallel sensorimotor streams, so that when simultaneous and compatible, they combine and produce greater motor activation in the brain, leading to improved ME (Bruton et al., 2020; Cisek & Kalaska, 2010; Eaves, Behmer, & Vogt, 2016). Alternatively, the visual guidance hypothesis argues that MI is the driver of AO + MI and the AO component simply acts as an external cue to guide MI (Chye et al., 2022; Meers et al., 2020; Vogt et al., 2013).

What is currently unclear is how similar the AO and MI components must be to achieve AO + MI congruency, as this is likely to vary according to the target action. Understanding this will be crucial for designing potential AO + MI-based therapies to ensure the greatest ME improvements in rehabilitative contexts. An example of this would be improvement of computer-based actions, such as keyboard typing, which can prove difficult for people with upper limb impairments, such as in Parkinson’s disease (Nes Begnum, 2010). Designing AO video materials that must *exactly* match the imagined and intended typing output would increase costs and reduce reusability. If a more general AO component that does not exactly match with the MI or intended action could have a similar influence on ME, this would be more feasible as a therapeutic tool. There is no research on whether AO + MI can influence execution of keyboard typing, though prior studies have shown motor-evoked potentials from finger muscles can be influenced differently by congruent, coordinative, and incongruent AO + MI (Bruton et al., 2020; Meers et al., 2020). In Bruton and colleagues (2020), coordinative AO + MI occurred when participants imagined performing the same effector movement as that observed, but with a different finger. In the context of keyboard use, observing typing of non-matching words during AO + MI would involve execution of very similar finger/hand movements, yet the actual content typed would be incongruent. Thus, it is unclear whether such an AO component would complement, or conflict with, simultaneous MI of target words.

This study investigated whether AO + MI can influence dexterous actions, such as keyboard typing, in a healthy adult population. Two experiments were conducted with the further aim of untangling whether a “general,” but not fully congruent, AO component would differently affect typing execution compared with “specific,” more congruent AO + MI.^1^ This would depend on how similar the “general” AO component and simultaneous MI component were perceived to be. If they were perceived as sufficiently similar, one would expect small or no ME differences compared with specific AO + MI, whereas if they were perceived as dissimilar, the components might conflict, reducing the effect on typing execution. This would have implications for developing future AO + MI-based rehabilitative programmes for patient populations with manual dexterity difficulties (e.g., Bek et al., 2021). To measure the influence of AO + MI on various motor aspects of physical typing, whole trial times, movement initiation speed, and typing speed were measured, as well as the number of typing errors made. Experiment 1 compared the short-term effect of general and specific AO + MI on typing execution across four different conditions. As Experiment 1 was conducted in a population expected to be proficient typists, we were unsure whether AO + MI would facilitate or impede participants’ typing. Therefore, we did not predict the direction of the AO + MI effect overall, but rather that specific AO + MI (AO + MIspec) would have a greater influence on typing execution compared with general AO + MI (AO + MIgen); i.e., should both AO + MI conditions facilitate typing execution relative to the baseline/control conditions, AO + MIspec would result in increased typing benefits relative to AO + MIgen, i.e., increased typing and movement initiation speed, and decreased whole trial times and typing errors. However, should both AO + MI conditions impede typing execution, we predicted that AO + MIspec would result in greater typing decrements compared with AO + MIgen. Experiment 2 repeated Experiment 1 but included an additional manipulation of stimulus speed to determine whether observed typing speed influenced ME. In addition, cognitive difficulty was increased in the control condition to evaluate whether cognitive effort influenced ME.

## Experiment 1

### Method

Both experiments were pre-registered on the Open Science Framework and can be accessed here: https://osf.io/re7xt and https://osf.io/2cb96. Adherence to transparency and open research methods are described in Supplemental material 2.

#### Participants

Fifty-one healthy University of Manchester students aged 18–26 years participated in this experiment (*M*_age_ = 20.2 years, *SD* = 1.6 years). A power analysis which assumed a medium effect size using a one-way analysis of variance (ANOVA) with four condition levels indicated 90% power with a sample of 50.^2^ The sample demographics are given in Table 1. Students participated in exchange for course credits or were offered a £10 shopping voucher. Participants were classified as “touch” typists if they reported that they typed with multiple fingers (*n* = 47)^3^ and “hunt-and-peck” (HP) typists if they typed with just their index fingers (*n* = 4). All participants had normal or corrected-to-normal vision and hearing. Participants gave informed consent online in questionnaire format before participating. Participants were not told the purpose of the experiment but were debriefed after the session. Ethical approval was granted by the University Research Ethics Council at the University of Manchester (2021-11118-19291).

**Table 1.** Participant demographics from Experiments 1 and 2.

Measure	Category	Experiment 1	Experiment 2
Gender, *n*	Female	42	14
	Male	8	3
	Other	1	3
Ethnicity, *n*	White	31	10
	Asian/Asian British	14	7
	Black	3	3
	Mixed	2	0
	Arab	1	0
Handedness, *n*	Right	41	16
	Left	6	3
	Mixed	4	1
Motor imagery (KVIQ-10), Mean (*SD*)	Visual	33.4 (6.1)	31.2 (7.8)
	Kinesthetic	26.0 (7.0)	28.2 (8.0)
Computer proficiency (CPQ-12), Mean (*SD*)	NA	27.7 (1.8)	26.7 (2.3)

*Note.* Values are either *n* of participants or mean (*SD*) as specified above. *SD* = standard deviation; KVIQ-10 = Kinesthetic and Visual Imagery Questionnaire; CPQ-12 = Computer Proficiency Questionnaire.

#### Materials and stimuli

This study was conducted online due to ongoing lab restrictions caused by the COVID-19 pandemic. Owing to this, a variety of computer software and equipment were used by participants to complete the experiment (see the online Supplemental material 3), and questionnaires were designed using Qualtrics (2020). The main typing experiment was built using PsychoPy 2021.1.4 and hosted online on Pavlovia so participants could complete the experiment in their web browser (Bridges et al., 2020; Peirce et al., 2019).

The typing videos in the main experiment depicted a first-person perspective of someone typing on a standard QWERTY keyboard with both hands. These were filmed using both touch and HP typing styles, with participants matched to the videos according to their self-reported typing style, such that, in the experiment, they saw an actor typing in a style similar to their own. In the videos for the AO + MIspec condition, the actor typed a single word 6 times amounting to a video of ~19 s. For the AO + MIgen videos, a generic sentence not including any target words was typed with the same number of key presses as the AO + MIspec videos. All videos were matched for typing speed at 120 BPM (whole word time: ~3.96 s, IKIs: ~499 ms), which was considered not too fast or slow, meaning that all videos were roughly the same length.

The words in the experiment were matched on a number of linguistic variables using LexOPS (Taylor et al., 2020). All words were six letters long and had a word frequency score (ZIPF) of 1.77–4 (Van Heuven et al., 2014), meaning all words were relatively low frequency and unlikely to be typed in daily life. This parameter was set to avoid ceiling effects in a sample expected to be proficient typists. Arousal, valence (G. G. Scott et al., 2019), age of acquisition (Kuperman et al., 2012), concreteness (Brysbaert et al., 2014), and imageability (Cortese & Fugett, 2004; Schock et al., 2011) were also controlled between conditions. Imageability and concreteness ratings were not available for a minority of selected words, but those without ratings were spread across all conditions. Overt action words and tool names were removed to prevent priming of actions through embodiment (e.g., Horoufchin et al., 2018), and similar words were also avoided to reduce practise effects or confusion. All words required both hands to be typed according to generic touch typing rules. A breakdown of the linguistic characteristics of the word sample is in Supplemental material 4.

#### Procedure

**Figure 1.**
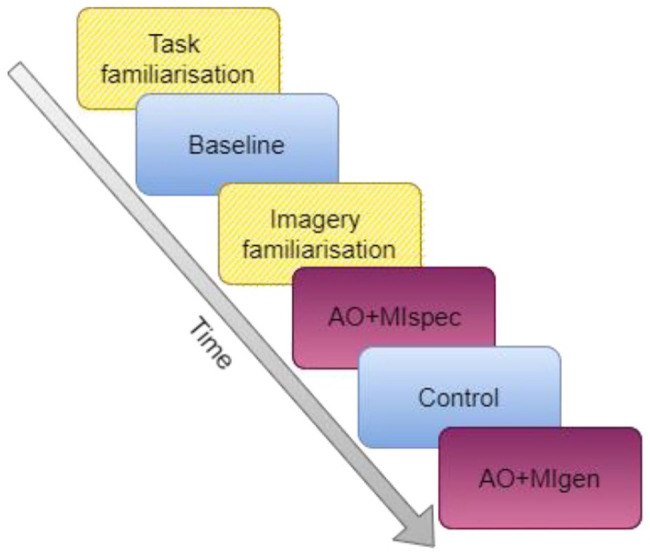
Sequence of familiarisation tasks and trial blocks of each condition in Experiment 1. *Note.* Yellow hatched blocks indicate when familiarisation tasks were completed. Solid blue blocks were held constant in time for all participants. Dark purple blocks were counterbalanced in order across participants (AO + MI conditions). Condition blocks (shown in blue and purple) contained 30 trials. Task familiarisation comprised three practice trials that could be repeated twice if necessary. Imagery familiarisation involved typing MI of a single word, repeated for 20 s.

All participants completed four conditions: baseline, control, AO + MIspec, and AO + MIgen. The baseline condition was always presented first and the control condition was always completed third in-between the AO + MI conditions. This enabled comparison between baseline and control conditions for analysis of practise/fatigue effects in the data. Furthermore, as the control condition was completed after imagery instructions had been given, there was an increased likelihood of spontaneous imagery occurring. Thus, comparing the control and baseline conditions also enabled examination of whether spontaneous imagery occurred (Vogt et al., 2013). The order of the two AO + MI conditions was counterbalanced across participants (see Figure 1). A total of 120 words were used in the experiment, of which 30 words were allocated to each condition. The allocation of words to condition was counterbalanced across participants, and the presentation of words within each condition was randomised.

Eligible participants completed an online questionnaire that included the 12-item Computer Proficiency Questionnaire (CPQ-12; Boot et al., 2015), the 9-item Patient Health Questionnaire (PHQ-9; Kroenke et al., 2001) to measure depression, as well as some questions on their demographics and computer use (see Supplemental material 5.1). Depression was measured to be included as a model covariate in the experiment analyses, due to depression being associated with impaired MI (Bennabi et al., 2014). Following completion of the questionnaire, the participant met the experimenter over Zoom and completed the Edinburgh Handedness Inventory (Veale, 2014), the 10-item Kinesthetic and Visual Imagery Questionnaire (KVIQ-10; Malouin et al., 2007), and the coin rotation task (Mendoza et al., 1995, 2009 see Supplemental material 6). The KVIQ-10 was adapted for online administration to measure the participant’s MI ability over Zoom.

**Figure 2.**
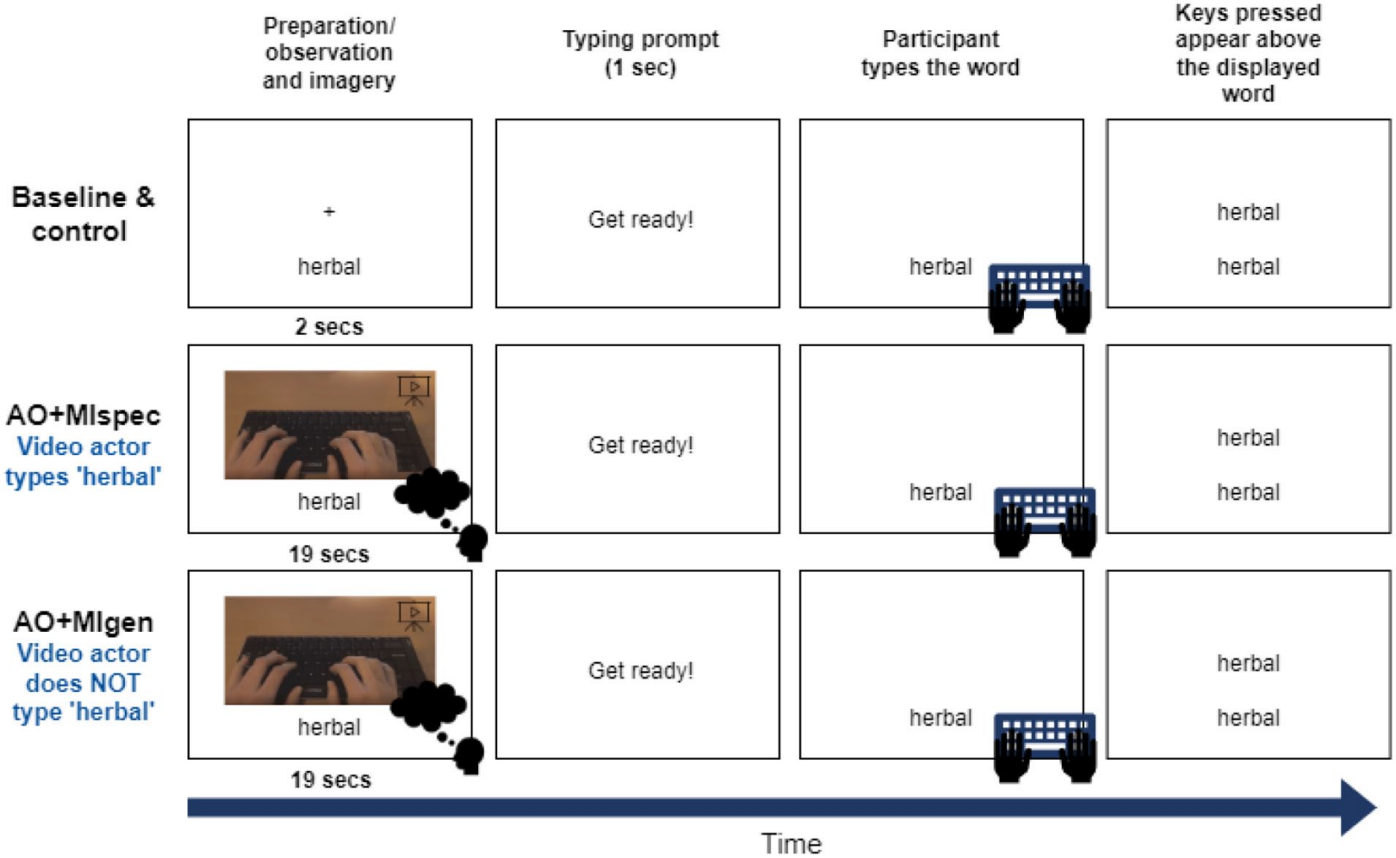
Procedure for each condition in Experiment 1. *Note.* Each horizontal row shows one trial from each condition. Different words were presented in each condition so that the participant only typed each word once—“herbal” is simply used here as an example. In both AO + MI conditions, the participant was instructed to imagine themselves typing the word on the screen (e.g., “herbal”) while watching the video.

The participant completed the main experiment in their web browser and used Zoom to share their screen with the experimenter so their progress could be monitored. The task required the participant to type a word displayed on their screen as quickly and accurately as possible after a typing prompt (see Figure 2). In the baseline condition, the participant was shown the target word with a fixation cross before the prompt was shown. After the prompt, the target word was displayed again, at which point the participant typed the word. When the participant had finished typing, they pressed the “return” key to submit their answer and continue to the next trial. Any keys that the participant pressed appeared in the centre of their screen and they were able to correct any errors using the “backspace” key. The control condition was identical to the baseline, except that different words were presented. In both AO + MI conditions, instead of just viewing the target word and fixation prior to the prompt, the participant watched a typing video with the target word displayed below. In the AO + MIspec condition, the video showed an actor typing the target word, whereas in AO + MIgen, the video actor typed non-matching words. While watching these videos, the participant was instructed to imagine themselves typing the target word. The participant was informed whether the typing in the video would match the target word or not at the start of each block (see Supplemental material 7 for full instructions). Practice trials were completed prior to each block except in the control condition (see Figure 1 and Supplemental material 8), and these trials could be repeated up to a maximum of 3 times where necessary to ensure understanding of the task. The participant also practised engaging in MI by imagining themselves typing the word “drawer” for 20 s, before proceeding to the first AO + MI block.

After each condition, the participant rated their typing performance on a Likert-type scale, with 1 meaning *very poor* and 5 meaning *very good*. To check whether spontaneous imagery occurred in the baseline and control conditions, the participant rated whether they had imagined, with 1 being *no, not at all* to 5 being *yes, all of the time*. After both AO + MI conditions, the participant rated their kinesthetic and visual imagery using the same scales as the KVIQ-10. After the main experiment, a final questionnaire was completed about the participant’s experience and imagery (see Supplemental material 5.2) before being debriefed and compensated.

#### Data processing and statistical analysis

Sequential analysis was performed with an interim “look” at the data after 25 participants. For further details on the interim analyses performed, see Supplemental material 9. We measured the time between trial onset and the “return” key being pressed to end the trial (whole word times) as a holistic measure of both typing speed and accuracy; the times between key presses (inter-key interval [IKI] times) as a measure of typing speed; and the time between trial onset and the first key press on each trial (first press times) as a measure of movement initiation speed. Accuracy was coded as the number of errors made on each trial, which were defined through manual coding according to set criteria to ensure consistency across participants (see Supplemental material 10). In addition, each key press was manually coded as correct or incorrect in binary and incorrect key presses were removed from analysis of IKIs and first press times. The “return” key press on each trial was also excluded from analysis of IKIs.

Participants’ data were excluded if they reported no imagery in either AO + MI condition (score of “1” on Likert-type scale), though this did not apply to any of the sample. Trials on which participants began typing too early (i.e., before or during the “Get ready!” prompt) were excluded from analysis (6.47% trials, *n* = 396), along with any trials manually noted as being invalid during participation (e.g., due to participants not paying attention or technical glitches, *n* = 5). Trials which had a first press time <100 ms or >10 s were removed from the first press times data, as this suggested either an incorrect measurement or instructions not being followed (0.63% trials, *n* = 36). No participants had more than one trial <100 ms which suggested instructions were followed. The “trimr” R package (Grange, 2018; Van Selst & Jolicoeur, 1994) was used to remove outliers using a non-recursive method at the level of condition for each participant for the speed measures (whole word time: 3.70% trials, *n* = 211; first press time: 2.83% trials, *n* = 159; IKIs: 2.88% observations, *n* = 797). Owing to the control condition not having any practice trials, separate analysis was conducted to ensure this would not affect the results (see Supplemental material 8).

Generalised linear mixed effects models (GLMMs) were conducted using the “lme4” R package (Bates et al., 2015) with the continuous dependent measures modelled to gamma distributions, and the model of accuracy data fitted to a negative binomial distribution. Each model included the fixed effect of condition, along with fixed covariates of PHQ-9 score, typing style, and condition order, which were allowed to interact with condition. Random intercepts of participant and word, as well as a random slope of condition × participant, were also included. In all models, the baseline condition was initially coded as the intercept. If there were no significant differences between the baseline and other conditions, the model was re-run with AO + MIspec as the intercept to reflect our primary comparison of interest to determine whether pairwise comparisons would be appropriate. All post hoc pairwise comparisons were Tukey-corrected and effect sizes were generated using Cohen’s *d* (Westfall et al., 2014). Adherence to model assumptions was visually inspected, of which none appeared to be severely violated.

Analyses of the self-reported ratings of typing execution and imagery in each condition are described in more detail in Supplemental material 11. Owing to the use of sequential analysis, *p* = .0482 and *z* = 1.6621 were used to determine statistical significance at the final look.

### Results

Owing to the small number of HP typists (*n* = 4), any significant effects relating to typing style are not reported due to unreliable inference. Models of typing accuracy are reported in Supplemental material 12.1 for the purpose of brevity, and due to few comparisons reaching statistical significance. Results pertaining to the model covariates (condition order and PHQ-9 scores) are in Supplemental material 13–14. All values are rounded to three decimal places.

#### Whole word time

**Figure 3.**
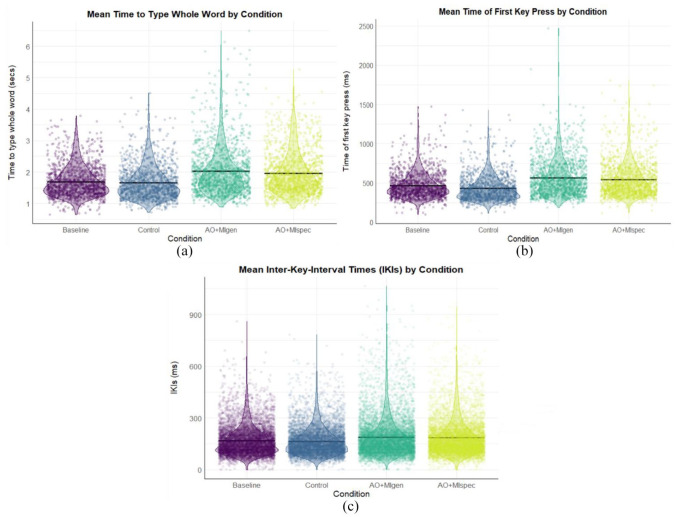
Descriptive statistics for whole word times, first press times, and IKIs in Experiment 1. *Note.* Black crossbars indicate the mean. The length of the violins shows the data distribution while the violin width shows the data density. Panel A displays whole word times across conditions; panel B displays first press times; and panel C shows inter-key interval times.

There was a significant difference between the AO + MIspec and control conditions (β = 0.772, *SE* = 0.094, *t* = −2.506, *p* = .012, *d* = 0.766). Pairwise comparisons (see Figure 3) revealed that whole word times were significantly shorter in the control condition (*M* = 1.65 s) compared with AO + MIspec (*M* = 1.99 s, *SE* = 0.051, *z* = 4.345, *p* < .001, *d* = 0.602), and significantly shorter in the control condition compared with AO + MIgen (*M* = 2.00 s, *SE* = 0.045, *z* = −3.516, *p* = .003, *d* = 0.625). Whole word times were also significantly shorter in the baseline (*M* = 1.71 s) compared with AO + MIspec (*SE* = 0.061, *z* = 2.921, *p* = .018, *d* = 0.494), and marginally shorter in the baseline compared with AO + MIgen (*SE* = 0.054, *z* = −2.492, *p* = .061, *d* = 0.516). There were no significant differences between AO + MI conditions (*p* = .999, *d* = −0.022).

#### First press times

There were significant differences between the baseline and control (β = −37.385, *SE* = 6.108, *t* = 6.108, *p* < .001, *d* = 0.282), AO + MIspec (β = 145.139, *SE* = 5.750, *t* = 25.243, *p* < .001, *d* = 1.090), and AO + MIgen (β = 106.400, *SE* = 6.566, *t* = 16.205, *p* < .001, *d* = 0.800) conditions. Pairwise comparisons (see Figure 3) indicated that first press times were significantly shorter in the baseline (*M* = 477 ms) compared with both AO + MIspec (*M* = 601 ms, *SE* = 11.58, *z* = −10.701, *p* < .001, *d* = 0.932) and AO + MIgen conditions (*M* = 598 ms, *SE* = 14.94, *z* = −8.132, *p* < .001, *d* = 0.914), and also significantly shorter in the control condition (*M* = 438 ms) compared with both AO + MI conditions (AO + MIspec: *SE* = 12.66, *z* = −12.871, *p* < .001, *d* = 1.225; AO + MIgen: *SE* = 13.94, *z* = −11.521, *p* < . 001, *d* = 1.207). In addition, first press times were significantly shorter in the control compared with the baseline condition (*SE* = 9.85, *z* = 3.960, *p* < .001, *d* = 0.293), likely due to a practise effect as there was no significant difference in spontaneous imagery ratings between these conditions (see Supplemental material 9.1). There were no significant differences between AO + MI conditions (*p* = .997, *d* = −0.018).

#### IKI times

IKIs differed significantly between AO + MIspec and the control condition (β = −0.169, *SE* = 0.064, *t* = −2.638, *p* = .008, *d* = 0.334), as well as a marginal difference between AO + MIspec and AO + MIgen (β = −0.101, *SE* = 0.054, *t* = −1.868, *p* = .062, *d* = 0.202), such that IKIs were slightly longer in AO + MIgen compared with AO + MIspec. Pairwise comparisons (see Figure 3) indicated participants had significantly shorter IKIs in the control condition (*M* = 168 ms) compared with AO + MIspec (*M* = 193 ms, *SE* = 0.032, *z* = 4.935, *p* < .001, *d* = 0.276) and AO + MIgen (*M* = 185 ms, *SE* = 0.028, *z* = −3.128, *p* = .010, *d* = 0.195), and IKIs were also shorter in the baseline (*M* = 175 ms) compared with AO + MIspec (*SE* = 0.039, *z* = 2.700, *p* = .035, *d* = 0.190). There was no significant difference between AO + MI conditions (*p* = .324, *d* = 0.081).

### Discussion

Results from Experiment 1 indicated that there were no significant differences between the specific and general AO + MI conditions in any measure of typing execution, which was contrary to our original hypothesis. This might suggest that different levels of AO + MI congruency recruit similar motor processes, as Eaves, Behmer, and Vogt (2016) identified no differences in event-related desynchronisation intensity in the sensorimotor or parietal areas between synchronised and static AO + MI.

Interestingly, we found that participants typed more slowly in both AO + MI conditions compared with the baseline and control conditions, across all speed-related measures. We consider three potential explanations for this finding regarding typing speed: a motor-cognitive interference effect; an automatic imitation effect; or a conflicting streams interference effect.

First, a motor-cognitive interference effect may have occurred due to the increased cognitive effort required to engage in AO + MI compared with the baseline and control conditions. Proficient typists, like the participants in this experiment, are likely to use automatic processes to execute habitual typing (Bannard et al., 2019; Rieger, 2004), but the AO + MI conditions forced participants to think consciously about their typing. This process may have engaged more executive processes and frontal neural networks than would have otherwise been engaged in habitual typing, such as the SMA, and disrupted typing execution in line with the constrained action hypothesis (Wulf et al., 2001). In addition, the motor-cognitive model posits that additional executive resources are required for MI compared with ME (Glover & Baran, 2017; Martel & Glover, 2023). Thus, the employment of these additional neural resources during AO + MI may have resulted in slower typing execution.

Second, an automatic imitation effect may have resulted in slower ME following AO + MI. Automatic imitation occurs when an observer is influenced by an actor’s movement, even if this is contrary to their movement goal (Gowen & Poliakoff, 2012; Heyes, 2011). The actor in the videos shown in the AO + MI conditions typed at a slower speed than participants in the baseline condition, so although participants were instructed to type as quickly and accurately as possible, observing an actor type slower than their habitual rate may have reduced their movement speed.

Third, it is possible that the AO and MI action representations elicited by the AO + MI conditions were incongruent and conflicted with one another, resulting in an interference effect and reducing activation of the brain’s motor areas. Indeed, many participants (70.59%) reported that the actor’s style of typing did not match their own (52.94% said the actor’s typing was worse than their own; 17.65% said it was better than their own). Participants identified as “touch” typists reported usually typing with multiple fingers on each hand, but this incorporated various typing styles, whereas the video actor typed according to standard touch typing rules, potentially resulting in incongruent AO + MI. Furthermore, as typing is a relatively small and fast movement, this may be a particularly difficult action to couple internal MI to an externally evoked AO component. The duration of a movement can be over-estimated during MI if the action is rapid and attention-demanding (Guillot & Collet, 2005), potentially causing MI to be slower than the faster AO component (Eaves et al., 2022). Alternately, as participants on average typed faster in the baseline condition than the video actor, it may be the case that the AO component was too slow and restricted ME (see Eaves et al., 2022), which may have also masked ME differences between specific and general AO + MI conditions.

To untangle these three explanations, Experiment 2 repeated Experiment 1 in a lab setting, with an added stimulus speed manipulation (fast vs. slow), as well as a more cognitively demanding random number generation (RNG) task added to the control condition. If the motor-cognitive interference account is correct and cognitive effort drove the slowed ME after AO + MI, we would expect no difference in typing execution between this more cognitively demanding control condition and AO + MI conditions, due to similar neural resources potentially being recruited by AO + MI and RNG tasks (see Martel & Glover, 2023). In addition, ME may be poorer in the control condition compared with the baseline if the effect is strong enough to counter any practise effects. If the automatic imitation account is correct, then we should see increased typing speed in the fast stimulus condition compared with slow in both AO + MI conditions, and participants should still type significantly more slowly in the slow AO + MI conditions compared with the slow-stimulus control condition. Finally, if the conflicting streams interference account is correct, typing should be more disrupted after both fast AO + MI conditions compared with slow AO + MI and control conditions because increasing the observed typing speed should increase the conflict of AO and MI streams due to MI becoming slower than the AO with increasing speed. We predicted that an automatic imitation effect would be the driving influence of our results due to the extensive literature documenting automatic imitation from AO (e.g., Gowen et al., 2016; Heyes, 2011).

## Experiment 2

### Method

#### Participants

Twenty healthy University of Manchester psychology students aged 18–24 years participated in this experiment (*M*_age_ = 19.2 years, *SD* = 1.7 years), who had not completed Experiment 1 (see Table 1 for demographics). Prior to data collection, a data simulation was conducted based on the findings from Experiment 1 based on the effect sizes from Aoyama and colleagues (2020), who also compared ME after AO + MI with different stimulus speeds. Data were simulated 100 times and with a sample of 20 participants, the results suggested this sample size would result in 100% power. The inclusion criteria were the same as Experiment 1, with the addition that all participants were required to be touch typists. All students participated in exchange for course credits.

#### Materials and stimuli

The experiment was completed on a Lenovo ThinkPad laptop attached to a Dell LCD monitor with a 20″ screen and an external standard QWERTY keyboard. The experiment was hosted locally using PsychoPy 2021.1.4. Questionnaires were designed using Qualtrics and completed online by participants both before and during the session.

The videos used for the slow stimuli condition were identical to Experiment 1 (see Experiment 1 Materials and stimuli), except that no HP videos were used as all participants were touch typists. The same videos as the slow condition were shown in the fast stimuli condition but played at 3× speed. Choice of video speed is explained in more detail in Supplemental material 15. The word stimuli were identical to Experiment 1 (see Experiment 1 Materials and stimuli).

#### Procedure

The two independent variables were condition (four levels) and stimulus speed (two levels). All conditions were the same as in Experiment 1 and the variable of stimulus speed applied to three of these conditions: control, AO + MIspec, and AO + MIgen. Equal numbers of fast and slow trials were presented in the relevant conditions, with the word stimuli displayed in fast versus slow trials counterbalanced between participants. Counterbalancing of the conditions and words presented in each condition was otherwise the same as in Experiment 1 (see Figure 1).

**Figure 4.**
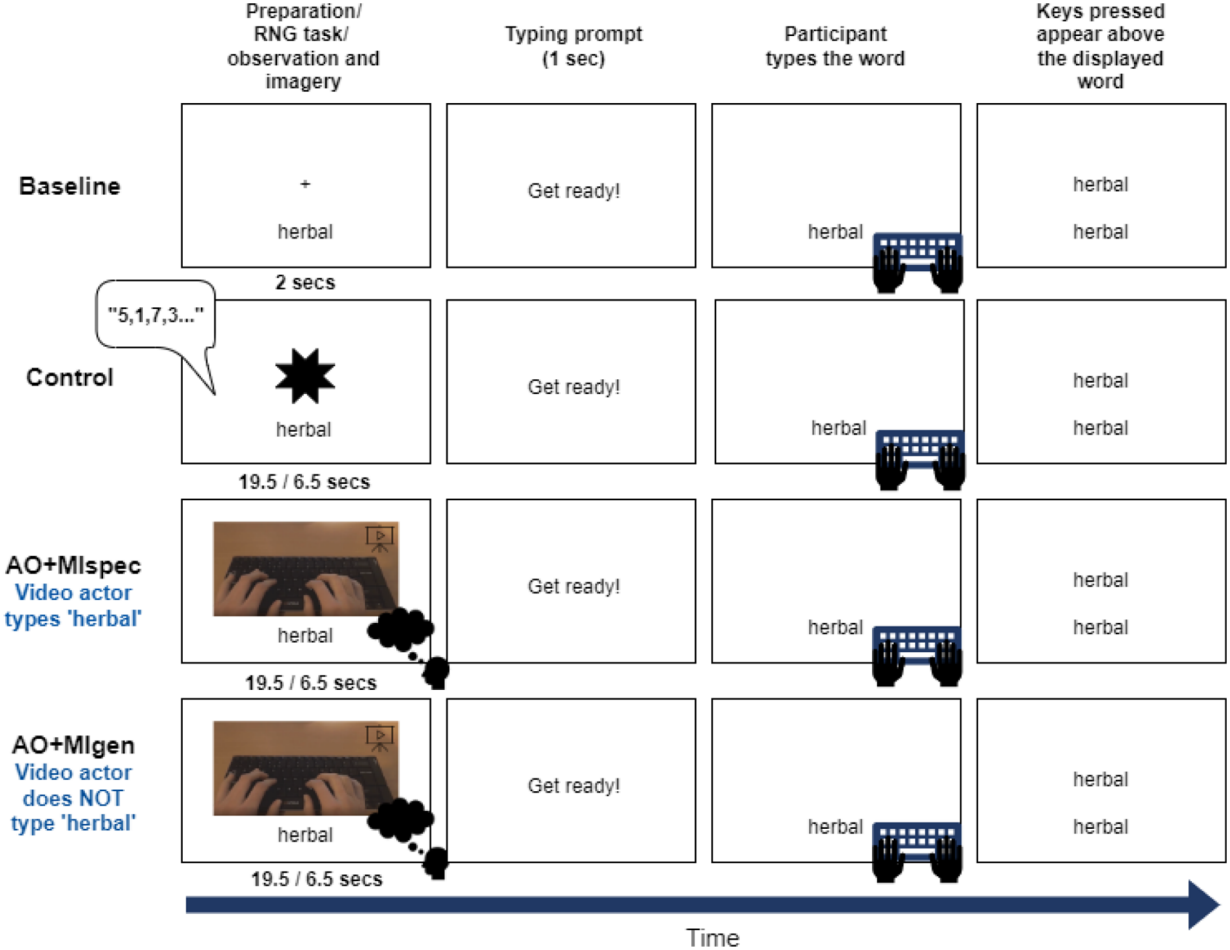
Procedure for each condition in Experiment 2. *Note.* Each horizontal row shows 1 trial from each condition. Different words were presented across conditions so that participants only typed each word once. In the control condition, participants verbally generated random numbers while the star was displayed. In both AO + MI conditions, participants were instructed to imagine themselves typing the word on the screen (e.g., “herbal”) while watching the video. In AO + MI and control conditions, 50% of trials were fast (19.5 s) and 50% were slow (6.5 s).

The measures administered were the same as in Experiment 1 except the coin rotation task was omitted. For the main experiment, the task was largely the same as Experiment 1, except in the control condition, the participant was shown a black star above the word stimulus. When the star was displayed on the screen, the participant was instructed to verbally generate random numbers, which were noted by the experimenter. Instructions for the RNG task were modified from those detailed by Baddeley (1966), as the original instructions relied on the participant imagining drawing numbers from a hat. As it was deemed critical to avoid imagery in the control condition, the instructions were modified to exclude this (see Supplemental material 7.3). In the fast stimulus condition, the star was displayed on screen for 6.5 s, and in the slow condition for 19.5 s to match the length of the fast and slow AO + MI videos, respectively. Once the star disappeared, the participant saw the usual “Get ready!” typing prompt and then typed the target word. In both AO + MI conditions, videos were either shown at the same speed as in Experiment 1 (slow trials) or sped up 3× faster (fast trials). The participant completed practice trials prior to each condition: three trials before the baseline and two trials before the other conditions (one practice trial of each stimulus speed). The procedure is shown in Figure 4.

#### Data processing and statistical analysis

Data were examined after the full sample had been achieved and statistical significance was determined by α = .05. As in Experiment 1, all participants reported experiencing imagery during at least one AO + MI condition. Trials were removed according to the same criteria as Experiment 1: if participants began typing too early (*n* = 62, 3.05% trials); if participants were not paying attention or due to technical errors (*n* = 5, 0.21% trials); and trials <100 ms or >10 s were removed from the first key press data (*n* = 5, 0.22% trials). No participants had more than one trial with first press times <100 ms. Outliers were removed using the same non-recursive method as Experiment 1 (whole word time: *n* = 84, 3.62% trials; first press time: *n* = 58, 2.55% trials; IKIs: *n* = 338, 3.02% observations).

The same GLMMs as Experiment 1 were used to compare differences between the four conditions collapsed across the factor of speed (Model 1), to see whether the pattern of results differed from Experiment 1. Two additional GLMMs were conducted for each dependent measure to compare conditions at each stimulus speed: one model excluded the baseline condition and included a fixed effect of stimulus speed to conduct a 3 × 2 analysis across control, AO + MIspec, and AO + MIgen conditions (Model 2) to assess the direct effect of speed on ME across conditions, and one which excluded the control condition and compared the baseline condition with the AO + MI conditions of each speed in a one-way analysis with five levels^4^ (Model 3) to compare typing after fast and slow AO + MI with baseline typing speed. In Model 2, an additional random slope of stimulus speed × participant was added if this resulted in a lower model AIC. If the same effects were found across multiple models, these were only interpreted in the first model they emerged to avoid inflating the Type I error. Models of typing accuracy were fitted to a Poisson distribution unless the model resulted in overdispersion, in which case a negative binomial model was used. In all models, the baseline condition was coded as the intercept, except for Model 2 which used the control condition, unless otherwise stated in the results. In addition, the RNG task was analysed using RGCalc (Towse & Neil, 1998) to determine whether participants followed instructions correctly and to inspect for outliers.

### Results

Results from the models of typing accuracy are reported in Supplemental material 12.2. Results from the RNG task and the model covariates (condition order and PHQ-9 scores), as well as relevant discussion, are included in Supplemental material 13–14 and 16.

#### Whole word times

**Figure 5.**
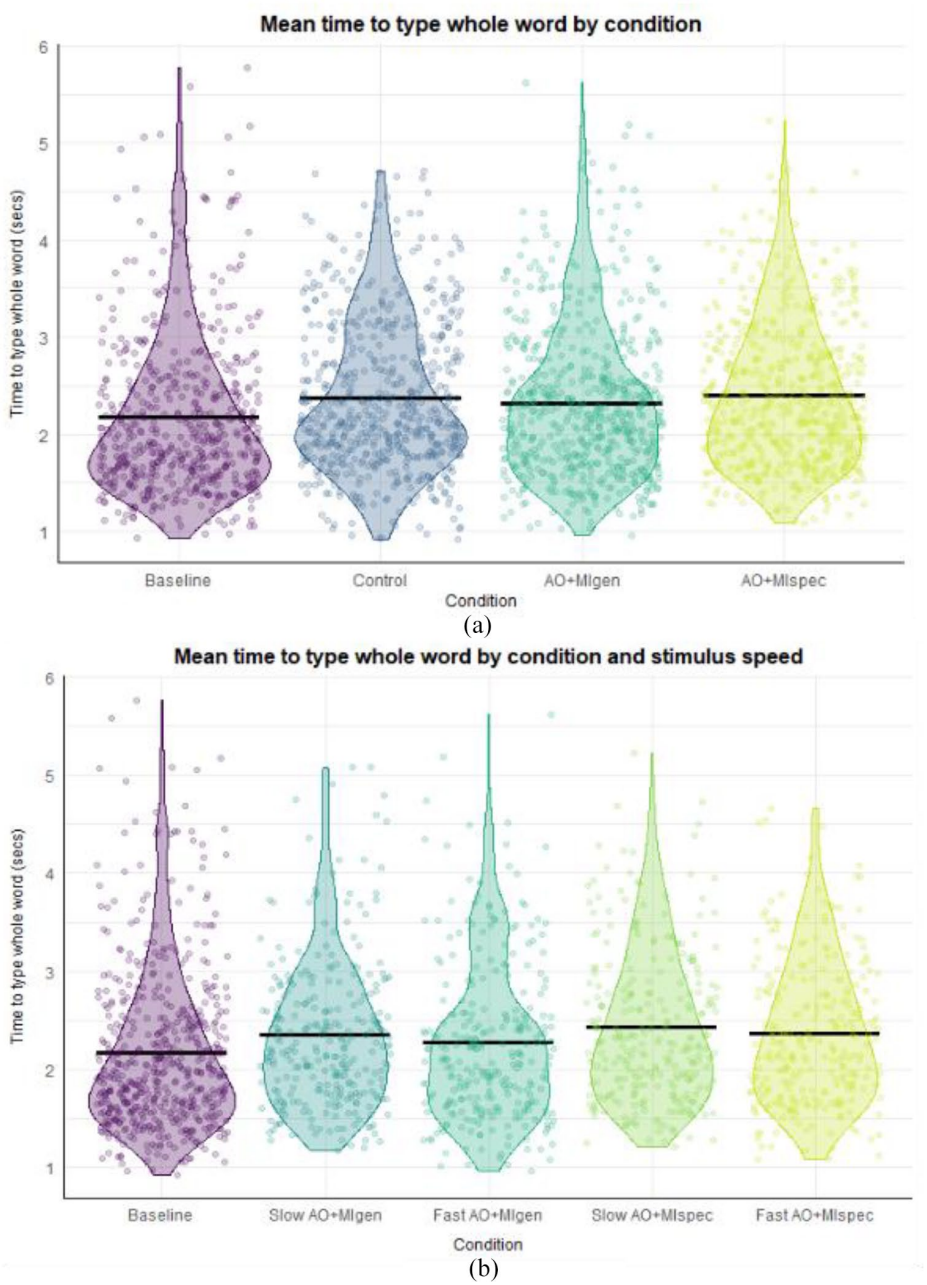
Descriptive statistics showing whole word times across conditions in Experiment 2. *Note.* Panel A. depicts descriptive data in line with Model 1 analysis, replicating the analysis conducted in Experiment 1 with conditions collapsed across the factor of stimulus speed; Panel B. shows descriptive data in line with Model 3, comparing baseline to AO + MI conditions of both speeds, excluding the control condition. In Model 3 participants were significantly faster in the baseline compared to fast and slow AO + MIspec, and slow AO + MIgen (*p* < .001; *d* > 0.321). Black crossbars indicate the mean. The length of the violins shows the data distribution while the violin width shows the data density.

In Model 1 (see Figure 5), there were no significant effects (*p* > .090, *d* < 0.282), and in Model 2, there was no significant main effect of stimulus speed, nor interaction with condition (*p* > .251, *d* < 0.182). Model 3 revealed a significant difference between baseline and the slow AO + MIspec condition (β = 0.087, *SE* = 0.044, *t* = 1.963, *p* < .050, *d* = 0.294) and the slow AO + MIgen condition (β = 0.145, *SE* = 0.043, *t* = 3.377, *p* < .001, *d* = 0.490). Pairwise comparisons of the main effect of condition in Model 3 (see Figure 5) revealed that whole word times were significantly shorter in the baseline (*M* = 2.09 s) compared with fast AO + MIspec (*M* = 2.32 s, *SE* = 0.019, *z* = −4.958, *p* < .001, *d* = −0.348); slow AO + MIspec (*M* = 2.37 s, *SE* = 0.021, *z* = −5.328, *p* < .001, *d* = −0.420); and slow AO + MIgen (*M* = 2.30 s, *SE* = 0.020, *z* = −4.240, *p* < .001, *d* = −0.321).

#### First press times

**Figure 6.**
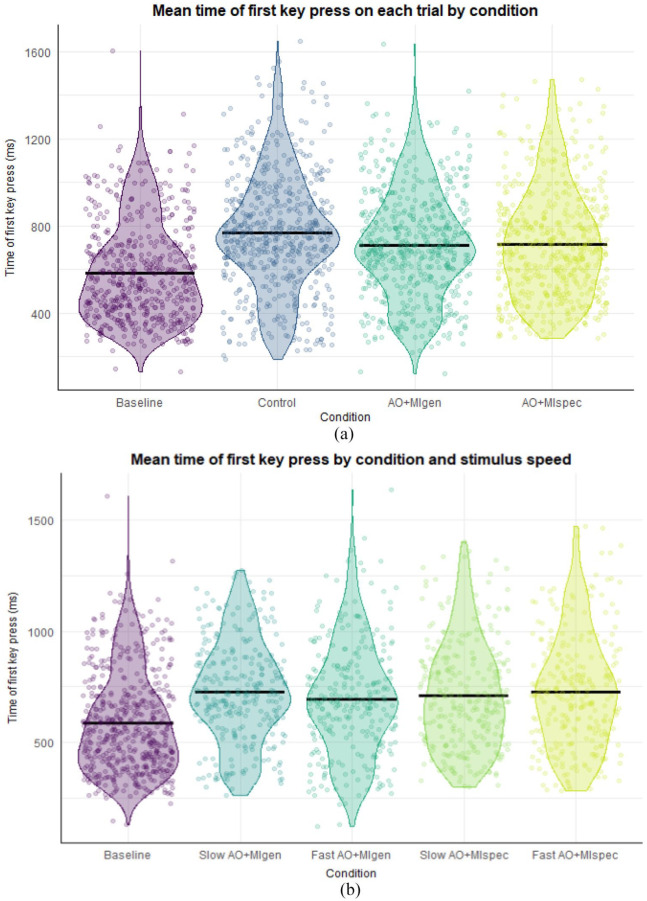
Descriptive statistics showing first press times across conditions in Experiment 2. *Note.* Black crossbars indicate the mean. The length of the violins shows the data distribution while the violin width shows the data density. Panel A shows first press times in each condition collapsed across stimulus speeds to reflect Model 1 findings, in which participants were significantly faster in the baseline condition compared to all others (*p* < .001, *d* > 0.620); Panel B reflects Model 3 findings comparing the baseline to AO + MI conditions of each stimulus speed, excluding the control condition. In Model 3 participants were significantly faster in the baseline compared to fast and slow AO + MIspec, and slow AO + MIgen (*p* < .036, *d* > 0.542).

Model 1 revealed significant differences between the baseline and control (β = 0.290, *SE* = 0.121, *t* = 2.388, *p* = .017, *d* = 0.861); baseline and AO + MIgen (β = 0.207, *SE* = 0.091, *t* = 2.270, *p* = .023, *d* = 0.614); and baseline and AO + MIspec conditions (β = 0.209, *SE* = 0.091, *t* = 2.308, *p* = .021, *d* = 0.621). Pairwise comparisons (see Figure 6) indicated that first press times were significantly shorter in the baseline condition (*M* = 548 ms, *SE* = 44.1 ms), compared with control (*M* = 730 ms, *SE* = 59.5 ms, *z* = −4.427, *p* < .001, *d* = −0.851); AO + MIgen (*M* = 676 ms, *SE* = 54.3 ms, *z* = −4.377, *p* < .001, *d* = −0.620); and AO + MIspec conditions (*M* = 686 ms, *SE* = 49.4 ms, *z* = −4.688, *p* < .001, *d* = −0.664).

In Model 2, there were significant interactions between AO + MIgen and stimulus speed (β = −0.160, *SE* = 0.067, *t* = −2.394, *p* = .017, *d* = −0.489), and between AO + MIspec and stimulus speed (β = −0.106, *SE* = 0.054, *t* = −1.970, *p* = .049, *d* = −0.352). Pairwise comparisons revealed participants had significantly shorter first press times in the fast AO + MIgen condition (*M* = 663 ms) compared with the fast control condition (*M* = 754 ms, *SE* = 0.043, *z* = 3.411, *p* = .009, *d* = 0.394) and first press times were shorter in slow AO + MIspec (*M* = 684 ms) compared with the fast control condition (*SE* = 0.0371, *z* = 2.87, *p* = .047, *d* = 0.296).

In Model 3 (see Figure 6), there were significant differences between the baseline and slow AO + MIspec (β = 0.194, *SE* = 0.091, *t* = 2.138, *p* = .032, *d* = 0.542); fast AO + MIspec (β = 0.217, *SE* = 0.103, *t* = 2.100, *p* = .036, *d* = 0.736); and slow AO + MIgen conditions (β = 0.276, *SE* = 0.106, *t* = 2.61, *p* = .009, *d* = 0.936). After Tukey-correction, the contrasts in Model 3 remained significant, such that participants had significantly shorter first press times in the baseline (*M* = 547 ms) compared with slow AO + MIspec (*M* = 674 ms, *SE* = 0.039, *z* = −4.416, *p* < .001, *d* = −0.587); fast AO + MIspec (*M* = 693 ms, *SE* = 0.043, *z* = −4.363, *p* < .001, *d* = −0.664); and slow AO + MIgen (*M* = 696 ms, *SE* = 0.043, *z* = −4.363, *p* < .001, *d* = −0.675). In addition, first press times were significantly shorter in the baseline compared with the fast AO + MIgen condition (*SE* = 0.044, *z* = −3.345, *p* = .007, *d* = −0.487).

#### IKI times

**Figure 7.**
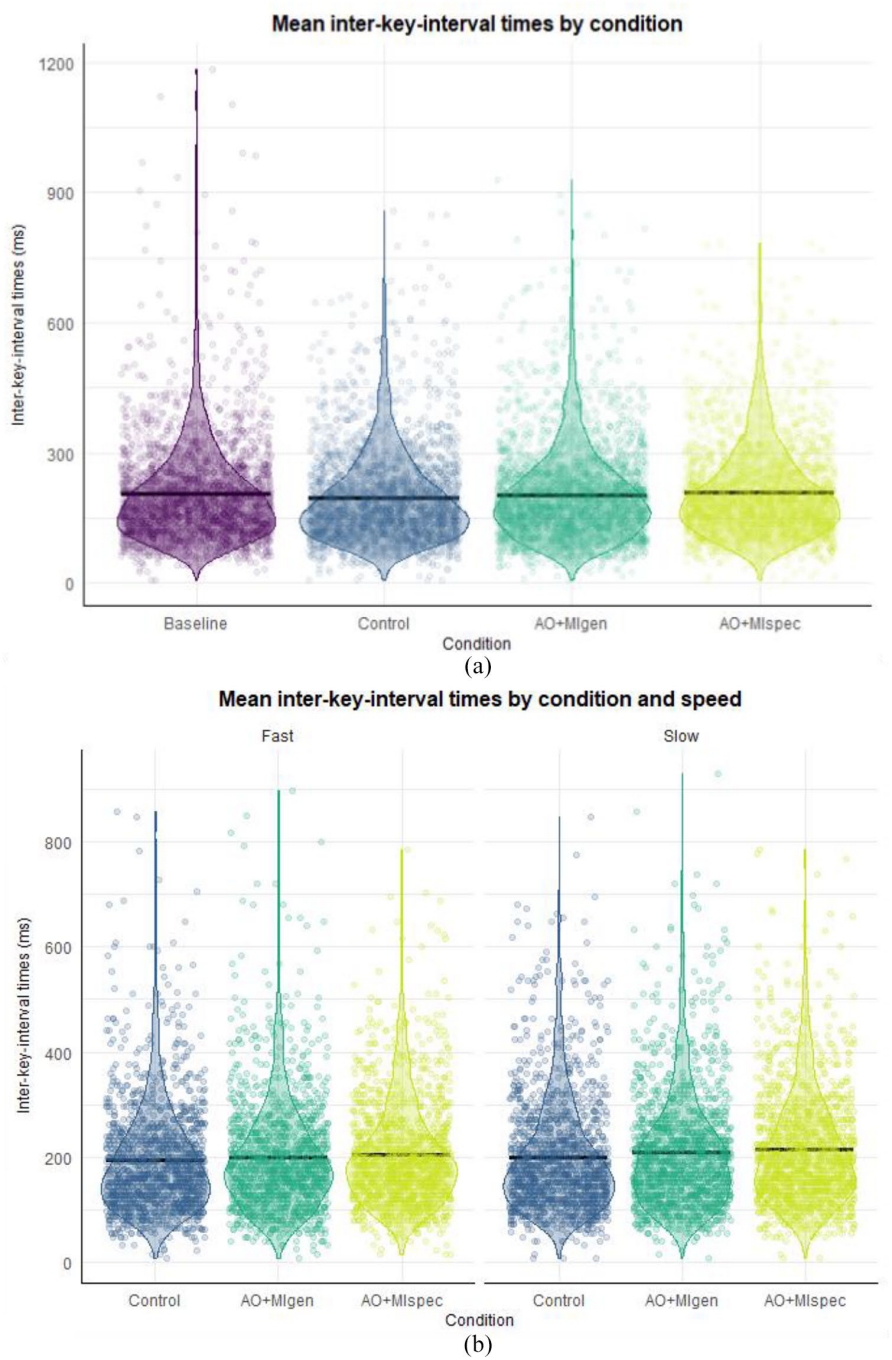
Descriptive statistics showing inter-key interval times across conditions in Experiment 2. *Note.* Black crossbars indicate the mean. The length of the violins shows the data distribution while the violin width shows the data density. Panel A shows Model 1 findings, replicating the analysis from Experiment 1 with conditions collapsed across the factor of stimulus speed; in which participants were significantly faster in the control condition compared to both AO + MI conditions (*p* < .019, *d* > 0.095); Panel B reflects Model 2 findings with a 3 (condition) X 2 (stimulus speed) design excluding the baseline condition. Participants were significantly faster in fast AO + MIgen compared to slow AO + MIspec (*p* = .004, *d* = −0.159).

Model 1 revealed no significant differences between the baseline and any other conditions (*p* > .115, *d* < 0.271). Owing to there being a significant interaction between condition and order (see Supplemental material 11.2.2), pairwise comparisons were conducted (see Figure 7), which indicated that IKIs were significantly shorter in the control condition (*M* *=* 189 ms, *SE* = 5.34 ms) compared with the AO + MIspec (*M* = 204 ms, *SE* = 0.0177, *z* = −3.857, *p* < .001, *d* = −0.154) and AO + MIgen conditions (*M* = 198 ms, *SE* = 0.015, *z* = −2.902, *p* = .019, *d* = −0.095).

In Model 2, there was a significant interaction between AO + MIgen and stimulus speed (β = 0.078, *SE* = 0.036, *t* = 2.193, *p* = .028, *d* = 0.167). Pairwise comparisons were conducted to explore the stimulus speed × condition interaction (see Figure 7). IKIs were significantly shorter in the fast control condition (*M* = 187 ms) compared with slow AO + MIgen (*M* = 202 ms, *SE* = 0.020, *z* = −3.636, *p* = .004, *d* = −0.163) and slow AO + MIspec (*M* = 209 ms, *SE* = 0.022, *z* = −4.596, *p* < .001, *d* = −0.237). IKIs were also significantly shorter in the slow control condition (*M* = 192 ms) compared with slow AO + MIspec (*SE* = 0.021, *z* = −3.749, *p* = .003, *d* = −0.183). In the slow AO + MIspec condition, IKIs were significantly longer than those in the fast AO + MIgen condition (*M* = 202 ms, *SE* = 0.019, *z* = −3.632, *p* = .004, *d* = −0.159) and were marginally longer than the fast AO + MIspec condition (*M* = 199 ms, *SE* = 0.018, *z* = −2.705, *p* = .074, *d* = −0.109).

Model 3 revealed no significant main effect of condition (*p* > .234, *d* < 0.108).

### Discussion

In Experiment 2, we found evidence for an automatic imitation effect influencing IKIs, as participants typed faster in the AO + MIgen condition after observing the fast stimuli compared with the AO + MIspec condition when observing the slow stimuli. Participants were also marginally quicker in the AO + MIspec condition after fast compared with slow stimuli. No evidence was found for a conflicting streams interference effect. If this had been the case, we would have expected slowed ME with increasing stimulus speed, and as effects were found inverse to this for IKIs, this provides greater evidence for an automatic imitation effect. This automatic imitation effect may partially explain the slowed typing execution seen after the slow AO + MI conditions in Experiment 2 and could also be applicable to the slowed ME seen after AO + MI in Experiment 1. Nevertheless, the effect sizes were small and participants still typed more slowly in the fast AO + MI conditions compared with baseline in Experiment 2. It is unlikely that the videos needed to show even faster typing as the video actor in the fast condition already had shorter IKIs (*M* = 166.25 ms) than participants at baseline (*M* = 205.27 ms). This suggests AO + MI is not effective at increasing typing speed even with faster AO stimuli.

Importantly, evidence was also found to support a motor-cognitive interference effect influencing whole word and first press times. Converse to Experiment 1, in the present experiment, there were no differences in whole word times between the control and slow AO + MI conditions, and first press times were significantly slowed in the control condition compared with the baseline and slow AO + MIspec conditions. This indicates the addition of the RNG task impeded participants’ movement initiation and that this effect was strong enough to counter any practise effect, such as that seen in Experiment 1.

## General discussion

In this study, we investigated whether specific (observing typing of the same word) and general (observing typing of different words) AO + MI would differently affect typing execution, and in Experiment 2, we explored why AO + MI resulted in slowed typing execution relative to the no observation/imagery conditions. In both experiments, no reliable differences in either typing speed or accuracy were detected between specific and general AO + MI. In Experiment 2, when cognitive effort was increased, participants had slower first key presses and trial times, indicating that switching from an effortful cognitive task negatively affected movement initiation. Furthermore, participants exhibited faster IKIs when the AO component was faster during AO + MI compared with when it was slower, indicating an automatic imitation effect influenced typing speed.

The absence of ME differences between specific and general AO + MI conditions may be due to the different AO components not being sufficiently different from one another. Although the actor typed a congruent word in the specific AO + MI condition, many participants reported using a different combination of digits in their normal typing, meaning that neither AO + MI condition involved fully congruent AO + MI. Owing to emerging literature demonstrating that incongruent AO + MI has less influence on ME than congruent AO + MI, if participants had watched videos of their own typing, or an identical typing style, this may have exerted a greater influence on typing execution (Bruton et al., 2020; Eaves et al., 2014). However, the ME similarities seen between specific and general AO + MI could have wide-reaching benefits when designing materials for rehabilitation and training programmes. This could make the design of an AO + MI programme for improving typing execution significantly more cost- and time-effective with the use of a limited set of general videos as opposed to the need for multiple specific AO + MI videos. This training could be beneficial for populations such as people with Parkinson’s disease, who can struggle with computer typing due to their motor symptoms (De Wet, 2005; Hartikainen & Ovaska, 2015) or for people recovering from stroke (Hitchcock, 2015). However, more research is required to confirm whether these findings would translate to other movements.

In both experiments, participants had slower typing speeds after AO + MI compared with the baseline/control conditions. Experiment 2 revealed that movement initiation (first press times) and trial completion times (whole word times) were affected by increased cognitive effort, likely due to the demands of task-switching between the RNG/AO + MI task and typing execution (Kiesel et al., 2010). In an action-mode switching paradigm by Rieger and colleagues (2017), participants exhibited slower typing when switching between imagery and execution compared with blocks comprising solely execution trials. Thus, the design of the AO + MI conditions in the present experiments may have induced slower ME due to the nature of switching between action modes. However, increased cognitive effort and task-switching had less impact on IKIs. In the control condition in Experiment 2, IKIs were similar to Experiment 1 despite the addition of the RNG task. IKIs captured the time between key presses, which were likely moderated by more automatic processes than movement initiation times. Movement initiation may rely on more frontal resources than IKIs (Hikosaka & Isoda, 2010), meaning there would have been competing demands on frontal neural processes when executive resources were also required for the RNG/AO + MI tasks. In addition, once participants had switched to typing execution, there would likely not be ongoing movement costs after the first key press, which would further explain why IKIs appeared unaffected by task-switching demands.

Furthermore, it is interesting that the RNG task and AO + MI conditions had a similar influence on whole word times. Participants reported experiencing significantly less spontaneous imagery in the control condition (median = 1) compared with the baseline (median = 3; see Supplemental material 11.2), which indicated participants were less able to engage in imagery while completing the RNG task—together, these findings could suggest that similar cognitive and neural processes were engaged during the RNG task as during AO + MI, which is in line with the motor-cognitive model (Glover et al., 2020), though neurophysiological evidence would be required to confirm this.

In addition, a small automatic imitation effect influenced IKIs, as typing speed was quicker after the fast AO + MI conditions compared with slow in Experiment 2 (Heyes, 2011). Automatic imitation may not have affected whole word and first press times because the AO component emphasised the typing action itself, as opposed to typing initiation or error correction, which comprised these measures. Nevertheless, typing execution in the fast AO + MI conditions was still slower than that in the baseline and control conditions across all speed measures, which is not fully explained by either motor-cognitive interference or automatic imitation. That increasing the observed typing speed did not result in faster typing execution compared with baseline also indicates that the slowed typing execution following AO + MI was not due to the observed typing speed being too slow. While it could be the case that our samples of healthy young adults were too proficient at baseline to increase their speed as a result of AO + MI, it could also be that typing is too fast and complex of a movement to maintain accurate MI. O’shea and Moran (2018) similarly concluded that pianists were unable to effectively imagine fast, complex piano music due to insufficient attentional resources. However, all participants in this study self-reported MI in at least one AO + MI condition, which suggests participants were at least partially successful at imagining typing during the AO + MI conditions. Alternatively, as typing is largely an automatic movement, the act of directing conscious attention to the action, as occurs during MI, may disrupt the normal movement process (Wulf et al., 2001). Indeed, X. Zhang and colleagues (2021) found that internally focused attention during a finger-tapping movement (i.e., focusing on the fingers) resulted in greater movement disruption compared with when attention was focused externally. As it is the nature of MI to direct attention internally, it is possible that AO + MI and MI are less suitable strategies for improving automatic or habit-driven movements in healthy adults, relative to goal-directed movements, though further research directly comparing these different movement types is required. In contrast, AO + MI could be an effective strategy in patient populations with impaired habit-driven movements, such as in Parkinson’s disease (Bannard et al., 2019; Wu et al., 2015).

### Constraints on generality

A limitation of this research stems from the use of students as participants in both experiments, who were already proficient typists and so had less opportunity for typing improvements from AO + MI. The samples of students were chosen as, due to the lack of research on AO + MI of keyboard typing, as well as AO specificity during AO + MI, it was deemed more appropriate to conduct this novel research in a healthy young adult population. In addition, it is possible that greater imitation would be observed if participants used a keyboard identical to that shown in the AO + MI videos, or if all participants were 10-finger typists, who matched the typing style of the observed actor. However, the online nature of Experiment 1 reduced control over participants’ computer equipment, as well as the ability to confirm participants’ exact typing style.

## Conclusion

Specific and general forms of AO + MI resulted in comparable typing execution, with positive implications for developing more cost- and time-effective AO + MI-based training/rehabilitation programmes using more general AO stimuli. However, AO + MI resulted in slower typing execution compared with no imagery/observation. This was influenced by both increased cognitive effort in the form of task-switching, and automatic imitation, which should be considered when designing future AO + MI-driven training. Furthermore, the strong influence task-switching had on typing initiation adds further credence to the motor-cognitive model of MI (Glover & Baran, 2017).

## Supplemental Material

sj-docx-1-qjp-10.1177_17470218241241502 – Supplemental material for Stimulus specificity in combined action observation and motor imagery of typing Supplemental material, sj-docx-1-qjp-10.1177_17470218241241502 for Stimulus specificity in combined action observation and motor imagery of typing by Camilla Woodrow-Hill, Emma Gowen, Stefan Vogt, Eve Edmonds and Ellen Poliakoff in Quarterly Journal of Experimental Psychology

## Notes

1. This condition was named “specific” as opposed to congruent as individual differences in typing style would affect the congruency of the action observation (AO) component.

2. We over-recruited for this study by one participant relative to the planned sample in the pre-registration. This was because the additional participant started the study before the full sample had been reached so they were allowed to finish participating. The statistical models were compared with and without the extra participant’s data to see whether their inclusion altered the overall findings—as it did not, their data were included.

3. Note that participants were not required to be 10-finger typists.

4. This final model was not specified in the pre-registration but was crucial to understand whether increasing the video speed affected typing performance relative to participants’ typing at baseline.
