# Supplementary material for: Stimulus specificity in combined action observation and motor imagery of typing
Source: Q J Exp Psychol (Hove). 2024 Apr 10;78(3):575–93. doi: 10.1177/17470218241241502 (PMC11874476; doi:10.1177/17470218241241502)
Supplement: sj-docx-1-qjp-10.1177_17470218241241502 – Supplemental material for Stimulus specificity in combined action observation and motor imagery of typing [file sj-docx-1-qjp-10.1177_17470218241241502.docx]

Supplementary Material for:

**Stimulus specificity in combined action observation and motor imagery of typing**

**Authors:** Camilla Woodrow-Hill^1^, Emma Gowen^1^, Stefan Vogt,^2^ Eve Edmonds^1^ and Ellen Poliakoff^1^

^1^ Division of Psychology, Communication and Human Neuroscience, University of Manchester

^2^ Psychology Department, Lancaster University

**Author note:** Generality of the findings is constrained by the sample consisting of undergraduate students, though this was the most appropriate sample to test our hypotheses. This research was presented at the Research in Imagery and Observation conferences in 2022 and 2023. This work was pre-registered on the Open Science Framework (<https://osf.io/re7xt> and <https://osf.io/2cb96>) and all code and materials are made available here: <https://doi.org/10.6084/m9.figshare.24064686>

**Stimulus specificity in combined action observation and motor imagery of typing:**
**Supplementary materials**

Table of Contents

[2. Transparency and openness 3](#_Toc157175269)

[3. Participant computer equipment 3](#_Toc157175270)

[4. Linguistic characteristics of word stimuli 3](#_Toc157175271)

[5. Original questionnaires 3](#_Toc157175272)

[5.1. Computer-use questionnaire 3](#_Toc157175273)

[5.2. Post-experiment imagery questionnaire 6](#_Toc157175274)

[6. Coin-rotation task (CRT) 9](#_Toc157175275)

[7. Task instruction scripts 9](#_Toc157175276)

[7.1. Imagery instructions given to participants after the baseline condition 9](#_Toc157175277)

[7.2. Instructions given for the AO+MIspec condition (Experiment 1) 10](#_Toc157175278)

[7.3. Instructions for Random Number Generation task (Experiment 2 only) 10](#_Toc157175279)

[8. Analysis of trials in Experiment 1 control condition 11](#_Toc157175280)

[9. Sequential analyses 11](#_Toc157175281)

[10. Accuracy coding rules 12](#_Toc157175282)

[10.1. General 12](#_Toc157175283)

[10.2. Exceptions and specific cases 12](#_Toc157175284)

[11. Self-reported rating data 14](#_Toc157175285)

[11.1. Experiment 1 14](#_Toc157175286)

[11.2. Experiment 2 14](#_Toc157175287)

[12. Typing accuracy results 14](#_Toc157175288)

[12.1. Experiment 1 15](#_Toc157175289)

[12.2. Experiment 2 15](#_Toc157175290)

[13. Model covariate results 15](#_Toc157175291)

[13.1. Experiment 1 15](#_Toc157175292)

[13.1.1. First press times 15](#_Toc157175296)

[13.1.2. Inter-key-interval times 16](#_Toc157175297)

[13.2. Experiment 2 16](#_Toc157175298)

[13.2.1. Whole word times 16](#_Toc157175299)

[13.2.2. Inter-key-interval times 18](#_Toc157175300)

[13.2.3. Accuracy 19](#_Toc157175301)

[14. Model covariate discussion 20](#_Toc157175302)

[15. Choice of stimulus speed in Experiment 2 20](#_Toc157175303)

[16. Random Number Generation task 21](#_Toc157175304)

# Transparency and openness

Sample size determination, all data exclusions, measures and manipulations are reported in the manuscript. Journal Article Reporting Standards are followed (Kazak, 2018) and the present work was pre-registered on the Open Science Framework (https://osf.io/re7xt and https://osf.io/2cb96). Experiment data, source code and research materials are available on GitHub (https://github.com/CWoodrowHill/AO-MI-computerUse_studentExp). Pseudonymised personal data may be made available upon request. Data collection took place between 2021 – 2022.

# Participant computer equipment

In Experiment 1 most participants completed the experiment on a laptop with screen sizes ranging from 12-16”. Four participants had a separate monitor with a larger screen (~22”). Twenty-five participants completed the experiment on a Mac operating system (OS) and 26 used Windows OS. Participants were encouraged to use Google Chrome as their browser when completing the experiment but could also use Firefox or Edge depending on their preference. Safari was not compatible and could not be used.

# Linguistic characteristics of word stimuli

**Table 1**
Linguistic characteristics of word stimuli in Experiments 1 and 2

| Linguistic Variable | Mean(*SD*) | Source | Reference |
| --- | --- | --- | --- |
| No. phonemes | 5.17(0.78) | The CMU Pronouncing Dictionary | Weide (2014) |
| Word frequency | 3.06(0.49) | SUBTLEX-UK (Zipf score) | Van Heuven et al. (2014) |
| Age of acquisition (years) | 11.69(1.24) | NA | Kuperman et al. (2012) |
| Valence | 4.58(0.96) | The Glasgow Norms | Scott et al. (2019) |
| Arousal | 4.27(0.80) | The Glasgow Norms | Scott et al. (2019) |
| Concreteness | 3.10(0.95) | NA | Brysbaert et al. (2014) |
| Imageability | 3.88(1.09) | NA | Cortese & Fugett, (2004);  Schock et al., (2012) |

*Note.* All words were generated using LexOPS (Taylor et al., 2020) in R and were 6-letters long. Imageability and concreteness ratings were not available for a minority of selected words, but those without ratings were spread across all conditions.

# Original questionnaires

## Computer-use questionnaire

About you

1. What is your gender?
2. Female
3. Male
4. Non-binary
5. Other (please specify)
6. What is your ethnicity? (Taken from gov.uk website)

*White*

- 1. English, Welsh, Scottish, Northern Irish or British
  2. Irish
  3. Gypsy or Irish Traveller
  4. Any other white background

*Mixed or Multiple ethnic groups*

1. White and Black Caribbean
2. White and Black African
3. White and Asian
4. Any other mixed or Multiple ethnic background

*Asian or Asian British*

1. Indian
2. Pakistani
3. Bangladeshi
4. Chinese
5. Any other Asian background

*Black, African, Caribbean or Black British*

1. African
2. Caribbean
3. Any other Black, African or Caribbean background

*Other ethnic group*

1. Arab
2. Any other ethnic group

Your computer use

1. What type of computer set-up do you plan to complete the experiment on? E.g., if you will complete the experiment using a laptop computer but with a desktop monitor plugged in, please select 'desktop'
   1. Laptop
   2. Desktop

COMMENTS BOX

1. Please provide information on the computer and monitor you will be using to complete this study. If possible, please include the model, make and screen size. If you are not sure, look for labels/brand logos on your monitor and computer. These could be underneath your laptop or at the back of your computer/monitor.

An example of where to find this information on a computer monitor is below:

*<Monitor image here>*

COMMENTS BOX

1. Will you be using a Windows or Mac computer to complete the experiment?
   1. Windows
   2. Mac
   3. Linux or other

If other please specify

COMMENTS BOX

1. Does the computer you intend to use for the experiment have a built-in webcam?
   1. Yes, and I intend to use this for the study
   2. Yes, but I will use an external webcam instead
   3. No, I have an external webcam
   4. I do not have any kind of webcam
2. Do you have access to a tablet or smartphone with a built-in camera?
3. Yes
4. No
5. How often do you use a computer (meaning, in your current day-to-day life)?
   1. Daily
   2. 2-3 times a week
   3. Once a week
   4. 2-3 times a month
   5. Monthly
   6. Less than monthly

(If selected f) to above) What is the reason that you infrequently use a computer?

COMMENTS BOX

1. Do you have a dominant hand you usually type with?
   1. Right hand
   2. Left hand
   3. I use both interchangeably
   4. I use both simultaneously
2. Can you touch-type (i.e. type without looking at your hands)?
   1. Yes, I’ve taken a typing course before
   2. Yes, but I have never taken a course
   3. No
3. Which of the below best describes your current typing style?
   1. I use both hands to type and use multiple fingers (does not necessarily have to be all five)
   2. I use both hands to type, but only use my index fingers
   3. I use my left hand to type and only use my index finger
   4. I use my right hand to type and only use my index finger
   5. Other - COMMENTS BOX
4. Do you use any accessibility adjustments to your computer set-up to help you use a computer more easily? (E.g. different style of mouse/keyboard, arm/hand supports, changes to computer settings such as button sensitivity etc.)
   1. Yes
   2. No, I do not need them
   3. No, I was not aware of the options
   4. No, I have not gotten around to it/for other reasons
5. If you answered a) to the above, please provide details below of which accessibility adjustments you currently use below.

COMMENTS BOX

1. (If answered a) to above) Would you be willing to temporarily go without your accessible adjustments (e.g. for 1-2 hours)?
   1. Yes
   2. No

## Post-experiment imagery questionnaire

Verbal Questions (audio recorded through Zoom)

1. Could you tell me a bit about your experience completing this experiment?

2. Could you tell me about what you imagined when you were asked to do so, in this experiment?

3. Did you have any techniques or strategies to help you perform better in this experiment?

Written Questions (answered in Qualtrics)

1. When asked to imagine something, were you able to do this successfully?

a) Yes

b) No

c) Some of the time

1. How well were you able to imagine yourself typing on a keyboard? (1 = very poorly/not at all; 5 = very well/clearly)

TEXT BOX

1. When instructed to imagine something, which perspective did you tend to imagine from?

a) First-person (i.e. as though looking through your own eyes)

b) Third-person (i.e. as though observing yourself from outside your body)

c) Other

If you selected c) please provide more detail here:

COMMENTS BOX

1. Did you find yourself imagining something when you were not instructed to (whilst just seeing the words)?

a) Yes

b) No

If you answered a) please specify below what you were imagining

COMMENTS BOX

1. Which hand/s did you use to complete the typing tasks?

a) Right hand

b) Left hand

c) Both simultaneously

*d*) I switched hands during the task

1. How difficult did you find this experiment? (1 = very easy; 10 = very difficult/was not able to complete)

TEXT BOX

1. Did you notice that any of the sections improved your typing? (Select all that apply)

a) Just seeing the word

b) Imagining while watching a video of the same word being typed (keyboard only)

c*)* Imagining while watching a video of different words being typed (keyboard only)

d) None of the above

1. Did you notice that any of the sections in-particular made your typing worse? (Select all that apply)

a) Just seeing the word

b) Imagining while watching a video of the same word being typed (keyboard only)

c) Imagining while watching a video of different words being typed (keyboard only)

d) None of the above

1. How would you compare the actor’s ability in the videos to your own at typing?

a) Better than my own ability

b) The same as my own ability

c) Worse than my own ability

*d*) Not sure

1. How close did you feel with the person you saw typing in the videos? Please select the picture below that best describes your relationship *<Inclusion of Other in Self Scale (IOS; Zmyj et al., 1992). Answer options depicted in Figure 1.>*

*Note.* This was taken from the measure developed by Zmyj et al. (1992) and was included in the post-experiment imagery questionnaire as Q10 (see section 4.2).

**Figure 1.**

*Inclusion of the Other in the Self Scale: Participant Answer Options*


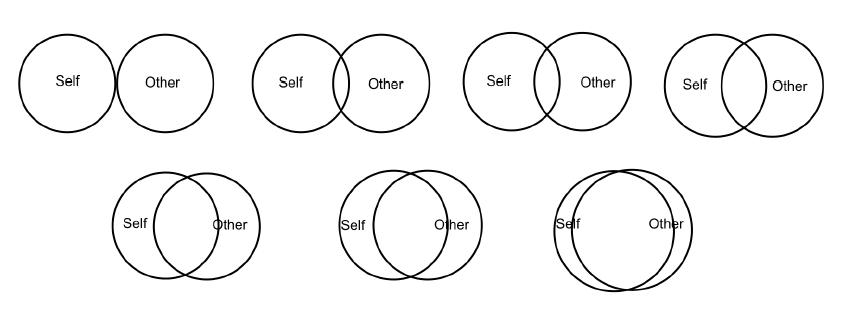


# Coin-rotation task (CRT)

The CRT is a measure of hand and finger dexterity which requires participants to rotate an American quarter between their thumb and fingers 20 times as quickly as possible while the time taken to perform this is recorded (Mendoza et al., 1995, 2009). This is repeated three times on each hand and an average (mean) time for each hand is calculated. Instead of using an American quarter, in Experiment 1 participants were asked to bring a British 10p coin to the Zoom session as this was most similar in size. In some cases participants were not able to bring a 10p coin and performed the task with different British coins. Participants performed 3 trials on their dominant hand first, followed by 3 trials on the non-dominant hand. If participants reported mixed handedness (*n* = 4) according to the Edinburgh Handedness Inventory short-form (EHI; Oldfield, 1971), the dominant hand was decided based on the majority of their handedness answers. For instance, if a participant reported on 75% of items that they ‘usually’ used their right hand, they would complete the CRT on their right-hand first. No mixed-handed participants reported an equal number of left- and right-handed EHI items. Across all participants, performance was better for the right hand (*M* = 17.90 secs, *SD* = 3.46 secs) than the left (*M* = 19.20 secs, *SD* = 3.90 secs), which was expected as most participants were right-handed (*n* = 41).

# Task instruction scripts

## Imagery instructions given to participants after the baseline condition

In the next sections of this experiment you may be asked to imagine yourself typing whilst you look at the word you need to type.

When I say ‘imagine’, what I mean is that I want you to think about how it would look and feel if you were typing the word yourself. For example, you might imagine that you can see your hands moving across the keyboard and pressing the keys. You might also imagine how it would feel to press those keys and move your hands in that way. You could think about the muscles and joints moving in your fingers, hands and wrists that you would use to type the word.

It is important that while you imagine this, you do not voluntarily move your hands. Keep your eyes open while you imagine.

I am going to give you 20 seconds to give this a go. Please rest your hands on your keyboard, but do not deliberately type anything. I would like you to imagine typing the word, "drawer". Keep imagining for the full 20 seconds.

## Instructions given for the AO+MIspec condition (Experiment 1)

In this section, your task will be the same as before - that is, to type the word you see on the screen AFTER the typing prompt. However, rather than just seeing the word before typing, you will also see a video of someone typing the same word you will need to type.

While you watch the video, imagine yourself typing the word on the screen. Imagine how it would LOOK to see your hands typing the word, as well as how it would FEEL to type it yourself. Think about the muscles moving in your hands and the feel of your fingers pressing the keys. While you watch the video and imagine this, rest your hands on your keyboard but DO NOT type until AFTER you see the typing prompt.

Remember to type as QUICKLY and ACCURATELY as possible^[[1]](#footnote-1)^.

## Instructions for Random Number Generation task (Experiment 2 only)

This section will be similar to the sections before - you will see a word on your screen which you must type AFTER the typing prompt.

However, this time the word will be displayed on the screen with a star for different lengths of time. Whenever you see this star you should say out loud random numbers between 1-10. The experimenter will make a note of the numbers you say. Try to make the numbers as random as possible and avoid number sequences. You may say numbers more than once. Try to say one number every second.

While you generate the numbers, rest your hands lightly on your keyboard and try not to move your hands. Do not start to type the word until AFTER you see the prompt. Type as QUICKLY and ACCURATELY as you can.

# Analysis of trials in Experiment 1 control condition

Due to the control condition not having any practice trials in Experiment 1, to determine whether early trials in the condition may have slower responses than later trials, the whole word time on the first 3 trials was compared to the remaining 27 trials in the control condition for each participant using a generalised linear mixed effects model (GLMM) fitted to a gamma distribution, which included random intercepts of participants and words^[[2]](#footnote-2)^. There was no significant difference found between the first 3, and later, trials (*SE* = .022, *t* = -1.081, *p* = .28), so all trials in the control condition were grouped together for the main analyses.

# Sequential analyses

As specified in the pre-registration, in Experiment 1 sequential analysis was performed which allowed an interim ‘look’ at the data after the first 25 participants, to determine whether data collection could be terminated early (Lakens, 2014; Lakens et al., 2021). An O’Brien and Fleming approximation was used for both efficacy and futility stopping rules (O’Brien & Fleming, 1979), the thresholds for which were estimated using the ‘gsDesign’ R package (Anderson, 2021). The test used an asymmetric two-sided design with non-binding futility bounds and assumed a 10% Type I error rate to generate both efficacy and futility thresholds. As there was an interaction between condition and condition order for the typing accuracy model which fell between these thresholds (*z* = 3.321; *p* = .020) at the interim look, data collection continued until the full sample of 50 participants was achieved. To reduce experimenter bias caused by observing the direction of results before data collection was terminated, the interim analysis was performed by a researcher not involved in the remaining data collection (Elsey et al., 2021).

# Accuracy coding rules

These are the rules used to manually code key presses in Experiments 1 and 2 for typing accuracy.

## General

- Mark correct key presses as ‘1’ and incorrect as ‘0’ in the AccuracyScore column
- Total the number of errors made for each word and mark the total in the AccuracyTotal column. Repeat the total for each row associated with that word/trial
- Mark ‘return’ key presses as ‘na’ in the AccuracyScore column but put the accuracy total score in the AccuracyTotal column
- Focus on key press order, not letter position, as this will inflate errors
- Mark the first time a key is pressed as correct or incorrect (in case a key is pressed twice after correcting an error) and the second press as incorrect
- The key press following ‘backspace’ must always be marked as 0 because typing is expected to be slower after an error correction

## Exceptions and specific cases

- If a couple of keys are pressed before the error is realised and backspace is pressed, mark however many keys are pressed before backspace as correct/incorrect accordingly. If a key/keys are pressed again after backspace, ignore the second press and mark as 0
- If correct keys are pressed but in the wrong order, mark both as incorrect regardless of whether these were corrected (e.g. CORETX instead of CORTEX) and score as 1 error. This only applies when 2 letters are switched, not more
- If someone tries to press a letter but gets it wrong twice (e.g. B-BACKSPACE-V-BACKSPACE-C), mark this as 2 errors
- Where a letter is missed and the following letters are in the correct order, mark the letters in the correct order as correct. If the error is corrected and the letters are pressed again, ignore the second presses. Score as 1 error
- If someone incorrectly presses backspace when the key before was actually correct, mark the first key press as correct and the same key pressed again after backspace as a 0. This would be 1 error
- If too many keys are deleted so they have to press a key again that they already pressed, mark the extra delete/s as an error
- If ‘BACKSPACE’ is pressed an incorrect number of times to correct their error, count an error for each correct letter they delete
- If an extra key is pressed, mark the extra incorrect key as ‘0’ and the correct one as ‘1’. Mark as 1 error.
- If a letter is missed, but then corrected by pressing backspace, mark this as an error but IF the additional letter does NOT immediately follow ‘backspace’, AND it is the first time they’ve pressed this letter, code the letter as ‘1’ . All keys following ‘backspace’ should be coded as ‘0’ (because presses following an error correction are likely to be slower (e.g., Q-W-R-BACKSPACE-BACKSPACE-U-A-R… for ‘quarry’ – the ‘A’ would be coded as ‘1’)
- If an incorrect key is pressed, which is a letter used later in the word, e.g. ‘S-U-R-E-F-E-R’ instead of ‘surfer’, mark both ‘E’ presses as ‘0’ and mark as 1 error. This is because having pressed the ‘E’ twice when there is only one in the word, ‘surfer’ could be considered additional practice and there may be an advantageous RT for the second press.
- If a key is pressed twice but it is clearly because the letter has not displayed on the screen (e.g., someone has typed fast enough that the letter did not show as having been pressed on their screen, and so they would naturally think they missed it and press it again), mark the extra press as ‘0’ but don’*t* include as an error
- If an extra key is pressed after ‘return’, but it is associated with the same trial (i.e., not the start of the next trial), code as ‘0’ and mark as one error, because this means the participant likely pressed this extra key while pressing the ‘return’ key

# Self-reported rating data

Participants’ self-reported ratings were analysed to see whether perceptions of their typing and imagery significantly differed in each condition to complement the main GLMM findings. Performance ratings were compared using a Friedman Test and effect sizes were generated using Kendall’s W. A post-hoc Nemenyi Test was used to compare ratings in each condition. Participants’ visual and kinaesthetic imagery ratings after the AO+MI conditions were compared using Wilcoxon Signed Rank Tests, as well as their presence of imagery ratings after the baseline and control conditions. All numeric values are rounded to 3 decimal places.

## Experiment 1

The Friedman Test comparing ratings of typing performance was significant (χ2 = 14.205; df = 3; *p* = .003; w = .093). The post-hoc Nemenyi Test revealed a significant difference between ratings in the control and AO+MIgen conditions (*p* = .026), whereby typing performance was rated as significantly higher in the control condition compared to AO+MIgen, in line with the objective measures of typing speed described in the main paper.

Wilcoxon Signed Rank Tests revealed no significant differences in participants’ visual or kinaesthetic imagery ratings between AO+MIspec and AO+MIgen conditions (*p* > .288). There was also no significant difference in spontaneous imagery ratings between the baseline and control conditions (*p* = .850).

## Experiment 2

A Friedman test was performed to compare participants’ self-reported ratings of their typing performance in each condition, but no significant differences were found (*p* = .358). Wilcoxon Signed Rank Tests were performed to compare visual and kinaesthetic imagery ratings in both AO+MI conditions, as well as spontaneous imagery ratings in the baseline and control conditions. No significant differences were found between imagery ratings in the AO+MI conditions (*p* > .235). However, participants reported significantly greater spontaneous imagery in the baseline condition (median = 3) compared to the control (median = 1) with a large effect size (*z* = -2.807, *p* = .005, *r* = .660). See main paper for further discussion.

# Typing accuracy results

## Experiment 1

The model output showed no significant effects (*p* > .712; see Table 2). It is likely that no significant effects were found for the accuracy measure due to ceiling effects as participants tended to be accurate typists making few errors overall.

**Table 2**
Output from the Model of Typing Accuracy in Experiment 1 with the AO+MIspec Condition Treated as the Intercept

| Contrast | *SE* | *z* | *p* | *d* |
| --- | --- | --- | --- | --- |
| Baseline / AO+MIspec | 0.423 | 0.369 | 0.712 | 0.212 |
| Control / AO+MIspec | 0.46 | -0.308 | 0.758 | -0.192 |
| AO+MIgen / AO+MIspec | 0.455 | -0.251 | 0.802 | -0.155 |

*Note.* The model used was a generalised linear mixed effects model fitted to negative binomial distribution. *d* refers to Cohen’s *d* as a measure of effect size.

## Experiment 2

Model 1 yielded no significant main effect of condition (*p* > .269, *d* ≤ .401). In Model 2 there was a significant interaction between stimulus speed and the AO+MIspec condition (*β* = -.707, *SE* = .346, *z* = -2.041, *p* = .041, *d* = -1.904), though pairwise comparisons revealed no significant effects after Tukey-correction (*p* > .877, *d* ≤ .560). In Model 3 there was also no significant main effect of condition (*p* > .411, *d* ≤ .394).

# Model covariate results

Only measures with significant covariate effects are reported below. For a description of the models conducted, see the main paper sections 2.1.5 and 3.1.4.

## Experiment 1


### First press times

Significant effects from the initial model output are shown in Table 3. After pairwise comparisons, the interaction between condition and order was only significant in the baseline condition. As the baseline was completed first by every participant and performance should, therefore, not be affected by order of the subsequent conditions, this result was deemed to be spurious and the statistics are not reported. There were no significant differences between AO+MI conditions in either order (*p* > .788).

**Table 3**
Significant Effects of Model Covariates from the First Press Times Model Output in Experiment 1

| Contrast | *β* | *SE* | *t* | *p* | *d* |
| --- | --- | --- | --- | --- | --- |
| Order | 23.613 | 5.716 | 4.131 | <.001** | 0.178 |
| Order × AO+MIspec | -13.139 | 6.035 | -2.177 | 0.029* | 0.099 |
| Order × AO+MIgen | -29.929 | 5.727 | -5.226 | <.001** | 0.225 |
| PHQ-9 × AO+MIspec | 4.930 | 1.724 | 2.859 | .004* | 0.037 |
| PHQ-9 × AO+MIgen | 7.090 | 2.154 | 3.291 | <.001** | 0.053 |

Exploratory Bonferroni-corrected pairwise comparisons of the PHQ-9 × condition trends were compared. There were significant differences between the trend in the baseline and AO+MIgen (*SE* = 2.15; *z* = -3.291; *p* = .006; *d* = .053); baseline and AO+MIspec (*SE* = 1.72; *z* = -2.859; *p* = .022; *d* = .037); and control and AO+MIgen (*SE* = 2.18; *z* = -2.970; *p* = .016; *d* = .049) conditions, with a greater positive relationship between PHQ-9 score and time of first key press in the AO+MIgen condition relative to baseline and control; and in AO+MIspec relative to control. These trends are shown in Figure 2.

*Note.* The model used was a generalised linear mixed effects model fitted to a gamma distribution. * denotes a significant effect at *p* < .0482; ** denotes an effect at *p* <.001. *d* refers to Cohen’s *d* as a measure of effect size. In this model the baseline condition was coded as the intercept.

### Inter-key-interval times

There was a marginal effect of the PHQ-9 (*β* = .007, *SE* = .004; *t* = 1.955; *p* = .051; *d* = .014). Bonferroni-corrected exploratory pairwise comparisons of the main effect of PHQ-9 score revealed a marginally significant positive trend in the AO+MIgen condition (*SE* = .004; *z* = 2.387; *p* = .068; *r* = .085), such that with increasing PHQ-9 score, IKIs in the AO+MIgen condition also increased.

## Experiment 2

Only models with significant covariate effects are reported below.

### Whole word times

In Model 2 there was a significant interaction between the AO+MIgen condition and the condition order (*β* = -.096, *SE* = .042, *t* = -2.270, *p* = .023, *d* = -.343), as well as a significant main effect of the PHQ-9 (*r* = -.195, *β* = -.011, *SE* = -.005, *t* = -2.101, *p* = .036, *d* = -.040), such that participants with higher depression scores were faster when typing whole words. Pairwise comparisons of the interaction between condition and order indicated that participants who completed AO+MIspec prior to AO+MIgen had faster whole word times in AO+MIgen (*M* = 2.19secs) compared to AO+MIspec (*M* = 2.37secs; *SE* = .021, *z* = -3.481, *p* = .007, *d* = -.285). In Model 3, there were significant interactions between PHQ-9 score and the fast AO+MIspec condition (*β* = .013, *SE* = .005, *t* = 2.691, *p* = .007, *d* = .042), as well as between the slow AO+MIgen condition and order (*β* = -.141, *SE* = .050, *t* = -3.818, *p* = .005, *d* = -.476). Comparisons of the interaction effect between condition and order of completion revealed that participants had significantly quicker whole word times in the baseline (*M* = 2.03secs) compared to slow AO+MIspec (*M* = 2.32secs, *SE* = .029, *z* = -4.031, *p* = .002, *d* = -.460); fast AO+MIspec (*M* = 2.31secs, *SE* = .027, *z* = -4.256, *p* < .001, *d* = -.436); and slow AO+MIgen (*M* = 2.39secs, *SE* = .028, *z* = -4.925, *p* < .001, *d* = -.559) when the AO+MIgen condition was completed first (see Figure 3). When AO+MIspec was completed first, participants were significantly quicker in the baseline (*M* = 2.15secs) compared to slow AO+MIspec (*SE* = .031, *z* = -3.302, *p* = .033, *d* = -.381). Bonferroni-corrected post-hoc comparisons of the interaction between PHQ-9 score and condition revealed a marginally significant difference in trends between the baseline and fast AO+MIspec conditions (*SE* = .005, *z* = -2.691, *p* = .055, *d* = -.042). There was a weaker negative correlation between PHQ-9 scores and whole word times in the fast AO+MIspec condition (*r* = -.0115) compared to in the baseline (*r* = -.256).


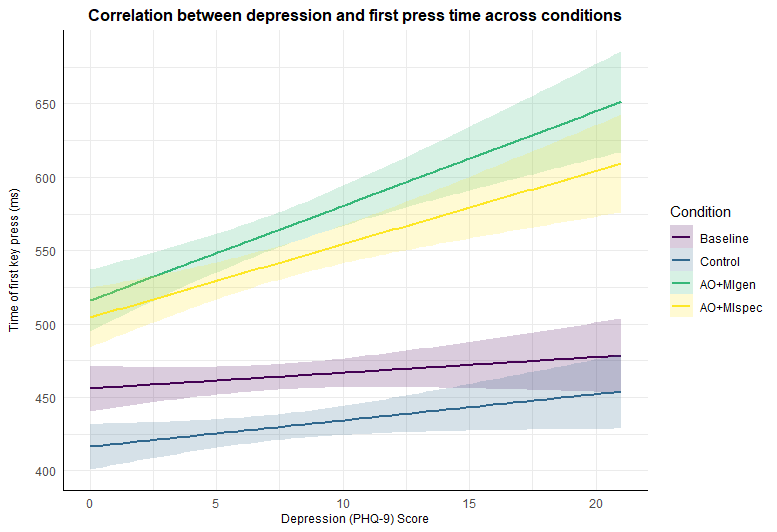


**Figure 2.**

*Trends Between Depression and First Press Times in Experiment 1*

*Note.* Lines indicate the linear relationship. Shaded regions show 95% confidence intervals.


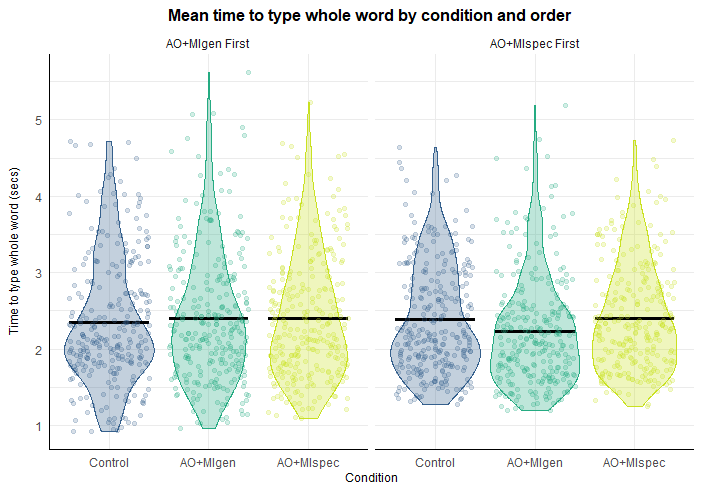


**Figure 3.**

*Descriptive Statistics Showing Whole Word Times Across Conditions in Experiment 2 by Condition Order, in Line with Model 2 Results*

*Note.* Black crossbars indicate the mean. The length of the violins shows the data distribution while the violin width shows the data density. Model 2 had a 3 X 2 design including the effect of stimulus speed and excluding the baseline condition.

### Inter-key-interval times

Model 1 revealed a significant interaction between AO+MIgen and the order of completion (*β* = -.085, *SE* = .042, *t* = -2.030, *p* = .042, *d* = -.179) and a significant main effect of condition order (*β* = .130, *SE* = .063, *t* = 2.058, *p* = .040, *d* = .271), such that participants were significantly quicker when completing the AO+MIgen condition first (*M =* 195ms, *SD* = 103ms) compared to when AO+MIspec was completed first (*M* = 213ms, *SD* = 117ms). There was also a significant main effect of the PHQ-9 (*r* = -.154, *β* = -.020, *SE* = .007, *t* = -2.939, *p* = .003, *d* = -.041), such that participants with higher depression scores had faster IKIs. Pairwise comparisons of the interaction with order showed that participants were only faster in the control condition compared to the AO+MI conditions when the AO+MIgen condition was completed first (see Table 4).

**Table 4**
*Pairwise Comparisons Showing Contrasts Between Control and AO+MI Conditions as an Interaction with Condition Order from the Model 1 Output of Inter-Key-Interval Times in Experiment 2*

*Note.* These comparisons were Tukey-corrected. Brackets show which AO+MI condition was completed first. * indicates which contrasts were significant at *p* < .05. *d* is Cohen’s *d* as a measure of effect size.

| Contrast | *SE* | *Z* | *P* | *d* |
| --- | --- | --- | --- | --- |
| Control (AO+MIgen) / AO+MIgen (AO+MIgen) | 0.021 | -3.878 | 0.003* | -0.184 |
| Control (AO+MIgen) / AO+MIspec (AO+MIgen) | 0.025 | -3.147 | 0.035* | -0.178 |
| Control (AO+MIgen) / AO+MIgen (AO+MIspec) | 0.049 | -2.383 | 0.25 | -0.277 |
| Control (AO+MIgen) / AO+MIspec (AO+MIspec) | 0.045 | -3.538 | 0.01* | -0.401 |
| Control (AO+MIspec) / AO+MIgen (AO+MIspec) | 0.023 | -0.109 | 1 | -0.005 |
| Control (AO+MIspec) / AO+MIspec (AO+MIspec) | 0.026 | -2.286 | 0.301 | -0.13 |

In Model 2, there was also a significant interaction between AO+MIgen and the condition order (*β* = -.084, *SE* = .033, *t* = -2.548, *p* = .011, *d* = -.179); as well as significant main effects of condition order (*β* = .135, *SE* = .057, *t* = 2.376, *p* = .018, *d* = -.289) and the PHQ-9 (*β* = -.013, *SE* = .006, *t* = -2.062, *p* = .039, *d* = -.028).

In Model 3 there was a significant interaction between fast AO+MIgen and the PHQ-9 (*β* = .009, *SE* = .004, *t* = 2.184, *p* = .029, *d* = .019). As in previous models, there were also significant main effects of the PHQ-9 (*β* = -.020, *SE* = .007, *t* = -2.938, *p* = .003, *d* = -.041) and condition order (*β* = .135, *SE* = .063, *t* = 2.134, *p* = .033, *d* = .279). In addition, there was a significant interaction between slow AO+MIgen and condition order (*β* = -.114, *SE* = .050, *t* = -2.271, *p* = .023, *d* = -.237). Pairwise comparisons of the condition × order interaction revealed no significant differences between baseline and AO+MI conditions in either order after Tukey-correction (*p* > .097). Bonferroni-corrected comparisons of the condition × PHQ-9 trends revealed no significant differences between conditions (*p* > .186).

### Accuracy

Model 1 indicated a marginal interaction between the baseline condition and the PHQ-9 (*β* = -.070, *SE* = .037, *z* = -1.903, *p* = .057, *d* = -.099). Bonferroni-corrected post-hoc comparisons of the condition × PHQ-9 trends revealed no significant differences in trends across conditions (*p* > .227).

In Model 2, there was a significant 3-way interaction between stimulus speed, the AO+MIspec condition and the PHQ-9 (*β* = .078, *SE* = .039, *z* = 2.004, *p* = .045, *d* = .211). Pairwise comparisons revealed no significant differences in trends between the PHQ-9 score and either condition or stimulus speed (*p* > .187).

In Model 3 there was a significant interaction between the AO+MIspec condition and the PHQ-9 (*β* = .119, *SE* = .040, *z* = 2.969, *p* = .003, *d* = .184). Bonferroni-corrected post-hoc comparisons revealed a significant difference in the condition × PHQ-9 trend between the baseline and fast AO+MIspec conditions (*SE* = .040, *z* = -2.969, *p* = .025, *d* = -.120). There was a small positive trend between PHQ-9 score and number of typing errors in the fast AO+MIspec condition (*r* = .100) while there was a negative correlation between number of typing errors in the baseline and PHQ-score (*r* = -.053).

# Model covariate discussion

Of the results highlighted above, one finding is of particular interest to the hypotheses. A significant difference in whole word times was found between AO+MI conditions *only* for participants who completed the AO+MIspec condition first. We suspect this is likely due to individual differences, as the participants completing AO+MIspec first ranked AO+MIspec as more difficult than AO+MIgen compared to participants who completed AO+MIgen first. The AO+MIspec-first group also scored notably lower on KI on the KVIQ-10 (mean = 23.9, *SD* = 8.09) compared to those who completed AO+MIgen first (mean = 32.4, *SD* = 5.32), suggesting group differences in motor imagery ability. It may be the case that participants with poorer imagery ability found it more difficult to couple the AO and MI components in AO+MIspec because, while the typed word was congruent, the style of typing and/or typing speed may not have been. Indeed, participants who completed AO+MIspec first were more likely to rate the actor’s typing as quite or very different to their own (*n* = 8) compared to participants who completed AO+MIgen first (*n* = 5). During AO+MIgen, as the participants knew the AO component would not match the target word and, thus, the MI component, participants may have been more able to ignore the AO component and focus on generating MI, which might explain these findings.

Regarding the effect of depression, while not of principal interest in these experiments, some conflicting results were found. In Experiment 1, there were positive relationships between depression and first press times, as well as IKIs in the AO+MI conditions; but in Experiment 2, negative relationships were identified between depression and whole word times, as well as IKIs. Thus, further research is warranted to untangle the relationship between depression and AO+MI.

# Choice of stimulus speed in Experiment 2

The decision to present videos that were 3X faster in the fast stimulus condition in Experiment 2 was made based on how much faster participants had typed in the baseline relative to the video actor in Experiment 1. Participants typed on average 2.85X faster than the video actor in the baseline condition. To determine whether simply increasing the playback speed of the existing videos would be perceived as naturalistic, an online Qualtrics questionnaire was administered to 12 healthy adults. Demographic data was not recorded. In the questionnaire, respondents watched typing videos of 8 different speeds: 1X, 1.25X, 1.5X, 1.75X, 2X, 3X, 4X, 8X, which were presented in random order. After each video, respondents were asked to rate how the typing speed compared to their own (1 = Much slower than my own; 10 = much faster than my own), and how naturalistic the typing appeared (1 = Very unnatural; 10 = Very natural). When rating the video speed, the median response for 2X speed was 5, indicating overall this speed was considered roughly the same as respondents’ own. For 3X speed, the median response was 7, suggesting this speed was slightly faster than respondents’ own. The most natural speed according to respondents was 2X speed (median = 7), with 3X speed rated second highest (median = 6). The decision to choose 3X speed over 2X was made because the greater the difference between the fast and slow stimulus conditions, the more likely performance differences would be seen in the data. In addition, as participants were almost 3X faster than the video actor in Experiment 1, displaying videos at 2X faster may have still displayed slower typing than our sample’s habitual rate.

# Random Number Generation task

Statistics for the task in both slow and fast conditions are presented in Table 5. As no participants emerged as consistent outliers across the various measures of randomness, no further analyses were conducted.

**Table 5**
Descriptive statistics of the Random Number Generation Task in Experiment 2

*Note.* Values given are the means with the standard deviation in brackets, rounded to 2 decimal places. Abbreviations are as follows: *n* = number of items produced in the sequence; RNG = Random Number Generation score at time lag 1; RNG2 = RNG but at time lag 2; TPI = Turning Point Index; RG = Repetition Gap. For a full description of these measures see Towse & Neil (1998).

| Measure | Speed | |
| --- | --- | --- |
|  | Fast | Slow |
| *n* | 5.15(1.14) | 15.45(3.14) |
| Adjacency | 20.56%(16.63%) | 21.05%(12.88%) |
| Redundancy | 31.26%(9.42%) | 7.70%(6.64%) |
| RNG | 0.00(0.03) | 0.11(0.13) |
| RNG2 | 0.00(0.00) | 0.08(0.11) |
| TPI | 101.53%(41.04%) | 95.67%(21.45%) |
| RG | 0.46(1.25) | 6.84(1.58) |

1. Instructions for the AO+MIgen condition were the same as the AO+MIspec condition except that participants were told they would “see a video of someone typing **different** words to what you must type”. [↑](#footnote-ref-1)
2. Note an ANOVA was not conducted as specified in the pre-registration due to the residuals not being normally distributed, making ANOVA an unsuitable statistical method. [↑](#footnote-ref-2)
